# Dog colour patterns explained by modular promoters of ancient canid origin

**DOI:** 10.1038/s41559-021-01524-x

**Published:** 2021-08-12

**Authors:** Danika L. Bannasch, Christopher B. Kaelin, Anna Letko, Robert Loechel, Petra Hug, Vidhya Jagannathan, Jan Henkel, Petra Roosje, Marjo K. Hytönen, Hannes Lohi, Meharji Arumilli, DoGA consortium, Hannes Lohi, Juha Kere, Carsten Daub, Marjo Hytönen, César L. Araujo, Ileana B. Quintero, Kaisa Kyöstilä, Maria Kaukonen, Meharji Arumilli, Milla Salonen, Riika Sarviaho, Julia Niskanen, Sruthi Hundi, Jenni Puurunen, Sini Sulkama, Sini Karjalainen, Antti Sukura, Pernilla Syrjä, Niina Airas, Henna Pekkarinen, Ilona Kareinen, Anna Knuuttila, Heli Nordgren, Karoliina Hagner, Tarja Pääkkönen, Antti Iivanainen, Kaarel Krjutskov, Sini Ezer, Auli Saarinen, Shintaro Katayama, Masahito Yoshihara, Matthias Hörtenhuber, Rasha Fahad Aljelaify, Fiona Ross, Amitha Raman, Irene Stevens, Oleg Gusev, Danika L. Bannasch, Jeffrey J. Schoenebeck, Katie M. Minor, James R. Mickelson, Cord Drögemüller, Gregory S. Barsh, Tosso Leeb

**Affiliations:** 1 Department of Population Health and Reproduction, School of Veterinary Medicine, University of California Davis, Davis, CA USA; 2 Institute of Genetics, Vetsuisse Faculty, University of Bern, Bern, Switzerland; 3 HudsonAlpha Institute for Biotechnology, Huntsville, AL USA; 4 Department of Genetics, Stanford University, Stanford, CA USA; 5 Dermfocus, University of Bern, Bern, Switzerland; 6 VetGen, Ann Arbor, MI USA; 7 Division of Clinical Dermatology, Department of Clinical Veterinary Medicine, Vetsuisse Faculty, University of Bern, Bern, Switzerland; 8 Department of Veterinary Biosciences, University of Helsinki, Helsinki, Finland; 9 Department of Medical and Clinical Genetics, University of Helsinki, Helsinki, Finland; 10 Folkhälsan Research Center, Helsinki, Finland; 11 Department of Veterinary and Biomedical Sciences, University of Minnesota, Saint Paul, MN USA; 12 Department of Veterinary Biosciences, University of Helsinki, Helsinki, Finland; 13 Department of Medical and Clinical Genetics, University of Helsinki, Helsinki, Finland; 14 Folkhälsan Research Center, Helsinki, Finland; 15 Department of Biosciences and Nutrition, Karolinska Institutet, Stockholm, Sweden; 16 Stem Cells and Metabolism Research Program, University of Helsinki, Helsinki, Finland; 17 Department of Biosciences and Nutrition, Karolinska Institutet, Huddinge, Sweden; 18 Science for Life Laboratory, Karolinska Institutet, Stockholm, Sweden; 19 Department of Veterinary Pathology, Faculty of Veterinary Medicine, University of Helsinki, Helsinki, Finland; 20 Department of Equine and Small Animal Medicine, University of Helsinki, Helsinki, Finland; 21 KFU-RIKEN “Translational genomics” Unit, RIKEN, Yokohama, Japan; 22 Roslin Institute, University of Edinburgh, Edinburgh, Scotland

**Keywords:** Evolutionary biology, Animal breeding, Genotype

## Abstract

Distinctive colour patterns in dogs are an integral component of canine diversity. Colour pattern differences are thought to have arisen from mutation and artificial selection during and after domestication from wolves but important gaps remain in understanding how these patterns evolved and are genetically controlled. In other mammals, variation at the *ASIP* gene controls both the temporal and spatial distribution of yellow and black pigments. Here, we identify independent regulatory modules for ventral and hair cycle *ASIP* expression, and we characterize their action and evolutionary origin. Structural variants define multiple alleles for each regulatory module and are combined in different ways to explain five distinctive dog colour patterns. Phylogenetic analysis reveals that the haplotype combination for one of these patterns is shared with Arctic white wolves and that its hair cycle-specific module probably originated from an extinct canid that diverged from grey wolves more than 2 million years ago. Natural selection for a lighter coat during the Pleistocene provided the genetic framework for widespread colour variation in dogs and wolves.

## Abstract (web summary)

Dogs exhibit remarkable variation in colour patterns. Here, the authors identify structural variants of independent regulatory modules for ventral and hair cycle expression of the *ASIP* gene that explain five distinctive dog colour patterns and trace back the origin of one colour pattern to an extinct canid.

## Main

A central aspect of the amazing morphologic diversity among domestic dogs is their colours and colour patterns. In many mammals, specific colour patterns arise through differential regulation of *Agouti* (*ASIP*), which encodes a paracrine signalling molecule and antagonist of the melanocortin 1 receptor (MC1R) that causes hair follicle melanocytes to switch from making eumelanin (black or brown pigment) to pheomelanin (yellow to nearly white pigment)^1–4^. In laboratory mice, *Asip* expression is controlled by alternative promoters in specific body regions and at specific times during hair growth and gives rise to the light-bellied agouti phenotype, with ventral hair that is yellow and dorsal hair that contains a mixture of black and yellow pigment^4^. Genetic variation in *ASIP* affects colour pattern in many mammals; however, in dogs, the situation is still unresolved, in large part due to the complexity of different pattern types, epistatic relationships with variants at other loci and challenges in distinguishing whether genetic association of one or more variants truly represents causal variation or just close linkage^5^.

Here we investigate non-coding variation in *ASIP* regulatory modules and their effect on patterning phenotypes in domestic dogs. We expand our analysis to include modern and ancient wild canids and uncover an evolutionary history in which natural selection during the Pleistocene provided a molecular substrate for colour pattern diversity today.

## Results

**Fig. 1:**
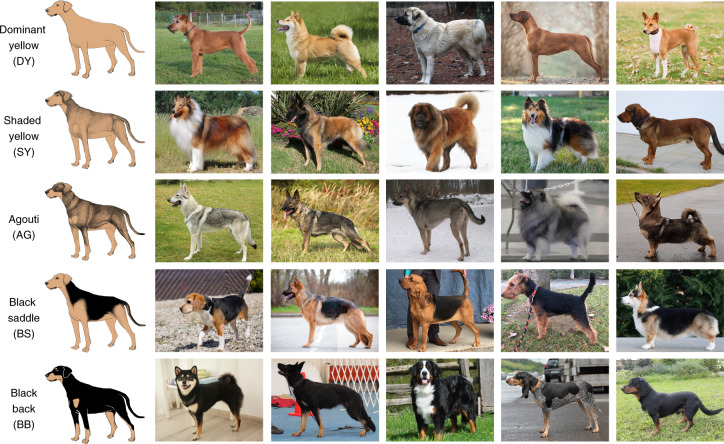
Coat patterns controlled by *ASIP*. Drawings of the five pattern types caused by *ASIP* regulatory variation are shown on the left, with representative photographs shown on the right. A completely black coat caused by homozygosity for *ASIP* loss-of-function is not shown. Within each pattern type, there may be variation due to other factors including: (1) the position of the boundaries between pheomelanic and eumelanic areas, for example in black saddle or black back; (2) the shade of pheomelanin (red to nearly white); (3) presence of a black facial mask or white spotting caused by genes other than *ASIP*; and (4) length and/or curl of hair coat. Patterns are displayed in order of dominance.

Expression of *ASIP* promotes pheomelanin synthesis; therefore, *ASIP* alleles associated with a yellow colour are dominant to those associated with a black colour. Although dominant yellow (DY) is common in dogs from diverse geographic locations, the most common coat pattern of modern wolves is agouti (AG)^6^, in which the dorsum has banded hairs and the ventrum is light. Three additional colour patterns are recognizable but all have been described historically by different, inconsistent and sometimes overlapping names that predate genomic analysis; we refer to these as shaded yellow (SY), black saddle (BS) and black back (BB) (Fig. 1 and Supplementary Table 1).

**Fig. 2:**
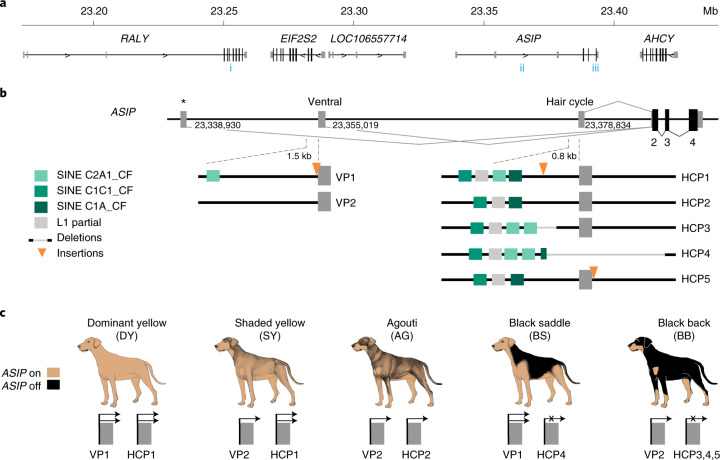
Structural variation at the *ASIP* locus in domestic dogs with different colour patterns. **a**, Genomic context (NCBI annotation release 105, CanFam3.1 assembly). Numbers in blue indicate previously reported variant associations—i (ref. ^16^), ii (ref. ^32^) and iii (ref. ^17^)—referred to in the Discussion and in Extended Data Fig. 2. **b**, The canine *ASIP* gene has three alternative promoters and 5′-non-coding exons (nucleotide coordinates denote their 3′-ends. Structural variation within 1.5-kb sections of the ventral- and hair cycle-specific promoters explains five different colour patterns in dogs. Two different VP haplotypes and five different HCP haplotypes are schematically indicated. The asterisk represents the third promoter and non-coding exon that is not related to *ASIP* pattern variation as described in the text and Fig. 1. **c**, Summary of how extended haplotype combinations are related to colour pattern phenotypes. Semiquantitative *ASIP* expression levels are depicted with one or two arrows or an X for no expression (Extended Data Fig. 1).

**Extended Data Fig. 1:**
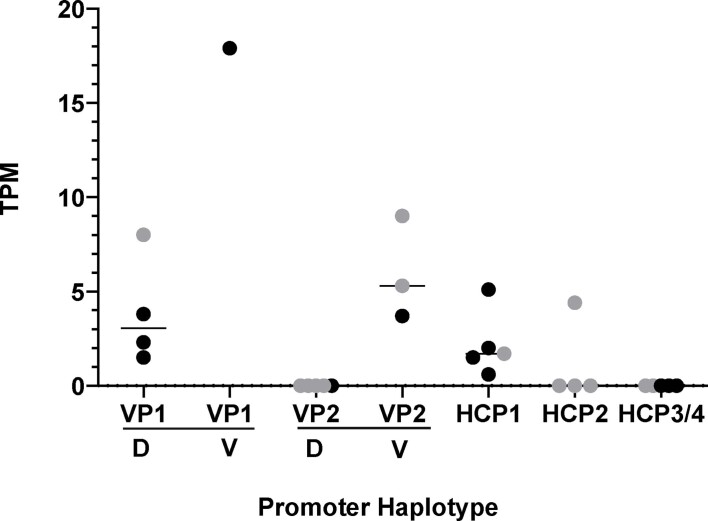
Relative transcription of promoter variants. RNA-seq counts (transcripts per million reads, TPM) from dorsal (D) and ventral (V) regions of two dominant yellow, three black back and four agouti dogs obtained from skin biopsies as described in Methods and in Supplementary Table 8. HCP samples also included a black saddle dog. Black dots are from RNA-seq data and grey dots are from STRT RNA-seq data.

We analysed skin RNA-sequencing (RNA-seq) data available from dogs of dominant yellow and black back patterns and identified three alternative untranslated first exons for dog *ASIP* (Fig. 2a, Extended Data Fig. 1 and Supplementary Table 2). As described below, two of the three transcripts vary in abundance between dominant yellow and black back dogs and the corresponding 5′-flanking promoters have sequence variation associated with dog pattern phenotypes. The 5′-flanking promoter regions for these two transcripts are orthologous to the ventral promoter (VP) and hair cycle promoter (HCP) in the laboratory mouse^4^; however, our genetic analyses (Fig. 2) reveal that the dog VP and HCP give rise to more complex patterns than their mouse counterparts. Transcripts associated with the third promoter, which lies ~16 kilobases (kb) upstream of the VP (Fig. 2b) did not vary in abundance in our dataset.

To better understand the relationship between promoter usage and pattern phenotypes, we inspected whole genome sequence data from 77 dog and wolf samples with known colour patterns (Supplementary Table 3). We used dogs that were homozygous at the *ASIP* locus to infer two VP haplotypes and five HCP haplotypes, consisting of multiple structural variants that lie within 1.5 kb of each transcriptional start site. VP1 contains a SINE element in reverse orientation relative to the transcription of *ASIP* and an A-rich expansion not found in VP2 (Fig. 2b left and Supplementary Table 1); the five HCP haplotypes differ according to the number and identity of SINE elements, all in the same orientation as *ASIP*, as well as additional insertions and deletions (Fig. 2b right and Supplementary Table 1). All structural variants were precisely delineated with Sanger sequencing.

**Fig. 3:**
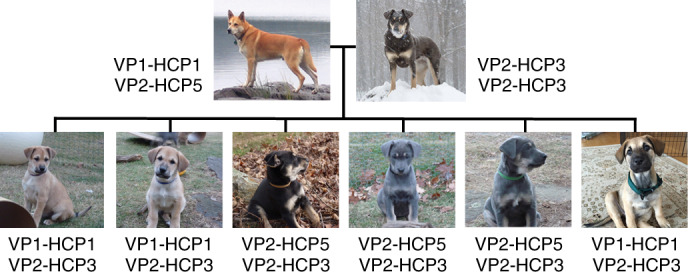
A family of dogs segregating dominant yellow and two black back haplotypes. Extended haplotype combinations in a family of Chinook dogs illustrating that, in combination with VP2, HCP3 and HCP5, both confer a black back phenotype.

**Extended Data Fig. 2:**
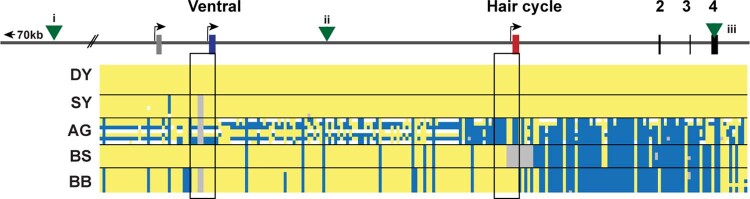
Dog haplotypes across the *ASIP* locus with comparison to previously associated variants for colour patterns. Dog coat pattern phenotypes are listed on the left. The genomic organization of the ASIP gene with its alternative promoters is illustrated at the top. Yellow indicates a homozygous match to the reference genome, grey deleted, white heterozygous and blue homozygous alternate allele. The black rectangles highlight the promoter regions. Green triangles represent the location of variants that were previously identified to distinguish different alleles for coat colour patterns: (i) The previously identified intronic duplication, “RALY dup”, associated with BS vs. BB haplotypes in some breeds, lies 86 kb to the left of the VP but recombinants (Supplementary Table 7) exclude a causal role for ASIP pattern variation^16^. Similarly, (ii), a SINE insertion associated with BB and BS haplotypes in some breeds and, (iii)^32^, missense variants in exon 4 associated with DY haplotypes^17^, are also excluded from a causal role in ASIP pattern variation by rare recombinants (Supplementary Table 7). In the samples presented here, the dominant yellow haplotype extends through the coding sequence where the missense variants associated with this haplotype were previously identified10. The results shown here will allow more accurate genetic testing in the future. Samples used are listed in Supplementary Table 3. Raw genotyping results are in Supplementary Table 4 and summary results comparing previously identified variants are in Table 1 and Supplementary Table 7.

These results were extended by developing PCR-based genotyping assays for the VP and HCP structural variants, examining their association with different pattern phenotypes in 352 dogs from 34 breeds and comparing these results to previously published variants (Table 1, Extended Data Fig. 2 and Supplementary Tables 1 and 4–7). As depicted in Fig. 2c and Table 1, diplotype combinations of VP1 or VP2 with HCP1, 2, 3, 4 or 5 are correlated perfectly with variation in *ASIP* pattern phenotype. For example, homozygotes for VP1-HCP1, VP2-HCP1, VP2-HCP2 are dominant yellow, shaded yellow and agouti, respectively (Supplementary Tables 4–7). Black saddle and black back dogs differ in their VP configuration but all carry HCP3, 4 and/or 5 in homozygous or compound heterozygous configurations. Because the level of *ASIP* activity is directly related to the amount of yellow pigment production, these genetic association results suggest that VP1 has greater activity than VP2, HCP1 has greater activity than HCP2 and HCP3, 4 and 5 all represent loss-of-function, since the HCP4 haplotype includes a large deletion of the hair cycle first exon (Fig. 2b) and fails to complement HCP3 or HCP5 (Fig. 3 and Supplementary Table 6). Importantly, increased activity from the ventral promoter (VP1 versus VP2) correlates with dorsal expansion of yellow pigment in black saddle compared to black back phenotypes (Figs. 1 and 2c), which indicates that the VP and HCP haplotypes function separately from each other.

**Table 1:** *ASIP* diplotype association with pattern phenotype

Pattern phenotype	Promoter diplotype	Concordant	Discordant
Dominant yellow (*n* = 114)^a^	VP1-HCP1 / VP1,2-HCP1,3,4,5	113	1^b^
Shaded yellow (*n* = 64)^a^	VP2-HCP1 / VP2-HCP1,3,5	52	12^b^
Agouti (*n* = 46)	VP2-HCP2 / VP2-HCP2,3,5	46	0
Black saddle (*n* = 53)	VP1-HCP4 / VP1,2-HCP3,4	53	0
Black back (*n* = 89)	VP2-HCP3,4,5 / VP2-HCP3,4,5	89	0

^a^Previous studies did not differentiate between the dominant yellow and the shaded yellow phenotype.

^b^These dogs had a eumelanistic masking pattern, which prevented reliable phenotype distinction between dominant yellow and shaded yellow.

The relationship between structural variation that delineates the different VP and HCP haplotypes and *ASIP* transcriptional activity was explored more directly in RNA-seq data from biopsies of dorsal and ventral dog skin (Supplementary Table 8 and Extended Data Fig. 1). Read counts from the RNA-seq data were consistent with expectations from the genetic association results: VP1 has greater transcriptional activity and is spatially broadened relative to VP2 (which is only expressed ventrally), HCP1 has greater transcriptional activity relative to HCP2 and no reads are detected from HCP3 or 4 (Fig. 2b and Extended Data Fig. 1). Taken together, these results provide a molecular explanation for *ASIP* pattern variation in dogs in which the VP and HCP function independently and for which structural variants in close proximity to VP and HCP modulate promoter activity.

**Fig. 4:**
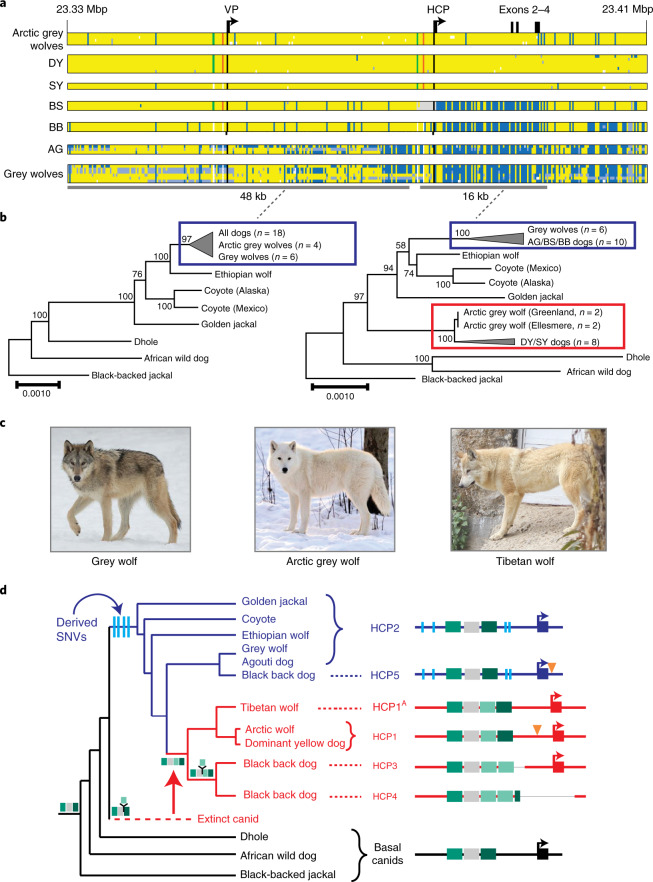
Yellow dogs and white wolves share an ancient HCP haplotype. **a**, Genotypes at 377 SNVs (columns) at the *ASIP* locus in grey wolves and dogs (rows), coded for heterozygosity (light blue), homozygosity for the reference (yellow) or the alternate (dark blue) allele or as missing genotypes (white). Alternate first exons (arrows) and nearby DY-associated structural variants (SINE insertions, green; polynucleotide expansions, orange) are included for reference. **b**, Maximum likelihood phylogenies, including seven extant canid species and the dog, from 48- and 16-kb intervals upstream or downstream of the HCP, respectively. Grey wolf/dog phyletic clades are highlighted with boxes to indicate relationships that are consistent (blue) or inconsistent (red) with genome-wide phylogenies. **c**, Images of a grey wolf, Arctic grey wolf and Tibetan wolf. **d**, A phylogeny representing distinct HCP evolutionary histories inferred from genetic variation in extant canids. Structural variants (as represented in Fig. 2) and derived SNVs (cyan) distinguish wolf-like canid (blue), ghost lineage (red) and basal canid (black) haplotypes.

Genetic relationships between variant *ASIP* regulatory modules were examined by comparing haplotypes in 18 homozygous dogs (for the structural variants at the VP and HCP and coding sequences) to those from ten contemporary grey wolves (Fig. 4a and Supplementary Table 9). Overall, agouti dog haplotypes were similar to those from grey wolves. However, dominant yellow and, to a lesser extent, shaded yellow dog haplotypes were similar to those from Arctic grey wolves from Ellesmere Island and Greenland, where all wolves are white (Fig. 4a–c). Notably, white coat colour in wolves represents pale pheomelanin, as in Kermode bears or snowshoe hares^7,8^. In the 64-kb segment that contains the VP, HCP and coding sequence, the Arctic grey wolf haplotypes are identical except for one polymorphic site (Fig. 4a, chr24: 23,337,523) and are distinguished from dog dominant yellow haplotypes by only six single nucleotide variants (SNVs) (Supplementary Table 10). Taken together, these observations suggest a common origin of dominant yellow in dogs and white coat colour in wolves without recent genetic exchange.

**Extended Data Fig. 3:**
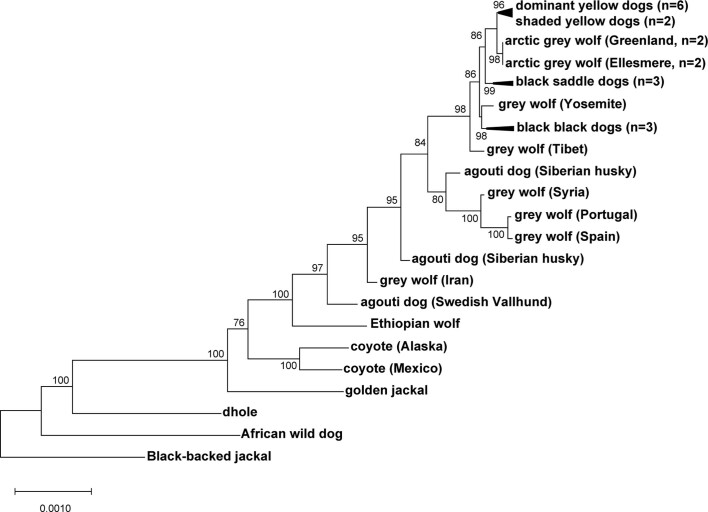
Expanded canid phylogenetic tree inferred from 48-kb region including the ventral promoter. An expanded version of the maximum likelihood tree shown in Fig. 4b, with 34 canids, representing 7 of 9 extant species.

**Extended Data Fig. 4:**
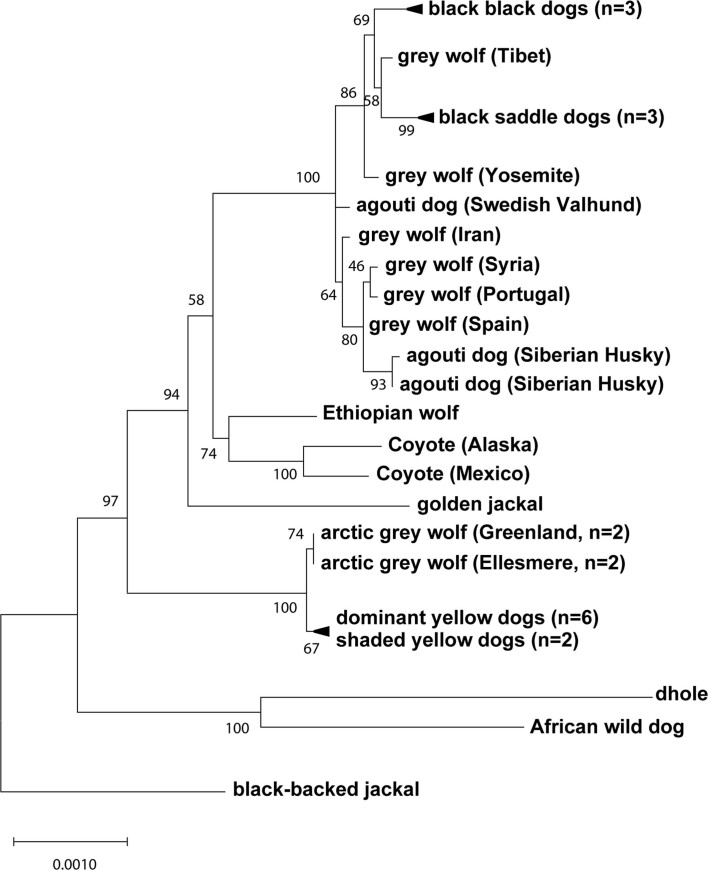
Expanded canid phylogenetic tree inferred from 16-kb region within and downstream of the hair cycle promoter. An expanded version of the maximum likelihood tree shown in Fig. 4b, with 34 canids, representing 7 of 9 extant species.

The evolutionary origin of *ASIP* haplotypes was explored further by constructing maximum likelihood phylogenetic trees for dogs, wolves and eight additional canid species (Supplementary Table 9). On the basis of differences in SNV frequency, the 48-kb VP segment was considered separately from the 16-kb HCP-exon 2/3/4 segment (Supplementary Information and Fig. 4a). In the VP tree, all dogs and grey wolves form a single clade, consistent with known species relationships^9^. However, in the HCP tree, the dominant yellow and shaded yellow dogs lie in a separate clade together with Arctic grey wolves; remarkably, this clade is basal to the golden jackal and distinct from other canid species (Fig. 4b and Extended Data Figs. 3 and 4).

**Fig. 5:**
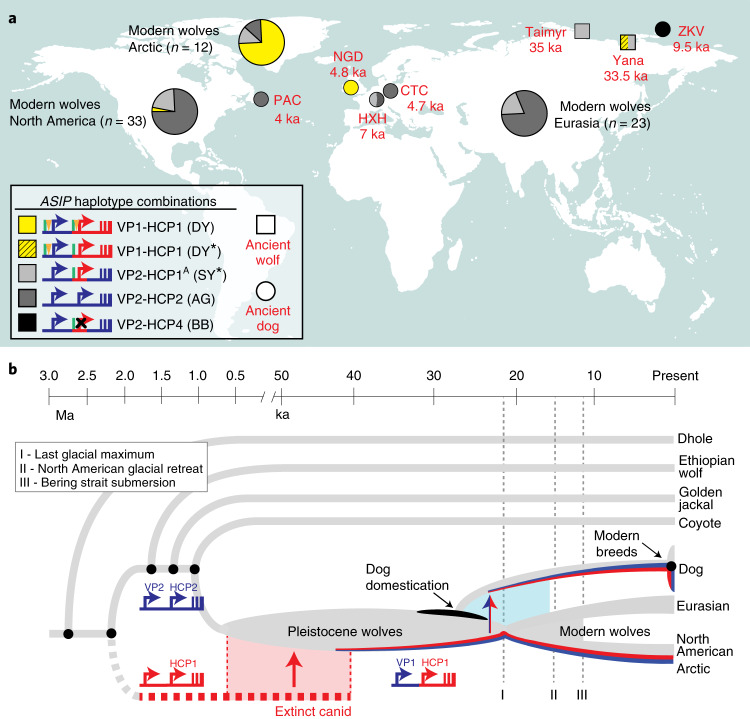
Distribution of *ASIP* alleles in ancient dogs and wolves, and an evolutionary model for dominant yellow acquisition. **a**, *ASIP* haplotypes were inferred from WGS of five ancient dogs (circles), two ancient wolves (squares) and 68 modern wolves (pie charts) distributed across the Holarctic (Extended Data Fig. 7 and Supplementary Table 12 show detailed haplotype representations). Asterisks indicate SY/DY haplotypes for which the HCP1 insertion is either absent (SY*) or not ascertainable (DY*). **b**, A model for the origin of the dominant yellow haplotype and its transmission into dogs and Arctic wolves, in which molecular alterations at modular promoters were acquired by introgression (red, HCP1) or by mutation in the grey wolf (blue, VP1). The timelines for speciation events, dog domestication and geological events affecting grey wolf dispersal are based on prior studies^10,33^.

**Extended Data Fig. 5:**
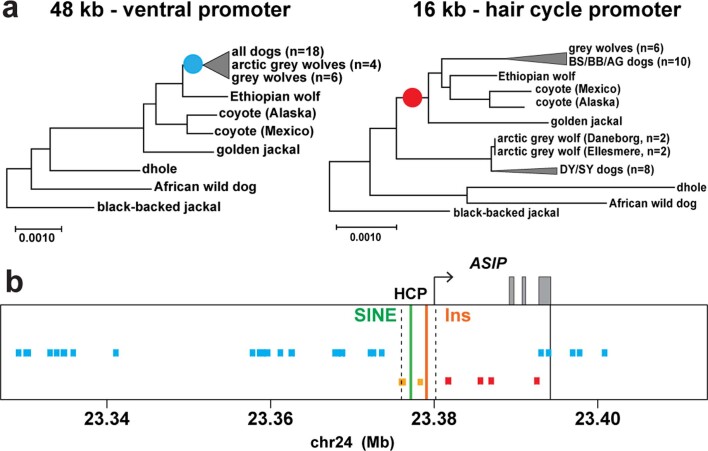
Genomic distribution of derived substitutions across the *ASIP* locus. **a**, Canid phylogenies for the ventral (48 kb) and hair cycle (16 kb) promoter regions, with relevant internal branches marked by the occurrence of derived variants plotted in (**b**). **b**, Derived substitutions shared by grey wolf and dogs (cyan). Ancestral alleles on DY/Arctic wolf haplotypes (red) or BB and DY/Arctic wolf haplotypes (orange) that correspond to derived substitutions among the core group of wolf-like canids (Supplementary Table 12). The broken lines demarcate the HCP region (chr24:23,375,800–23,380,000). The solid line signifies the downstream boundary for phylogenetic analysis. The solid green and orange lines indicate the positions of the SINE and 24 bp insertion, respectively, associated with the DY/Arctic wolf haplotype.

The pattern of derived allele sharing provides additional insight (Fig. 4d and Extended Data Fig. 5). As depicted in Figs. 2c and 4d, HCP2 is characterized by three small repeat elements that are shared by all canids and is therefore the ancestral form. In the branch leading to core wolf-like canids (golden jackal, coyote, Ethiopian wolf and grey wolf), there are nine derived SNV alleles within the HCP2-exon 2/3/4 segment (Extended Data Fig. 5 and Supplementary Table 11), four of which flank the repeat elements close to HCP2 (Fig. 4d and Extended Data Fig. 5). None of the nine derived alleles is present in the dominant yellow HCP1-exon 2/3/4 segment haplotype which also carries an additional SINE close to HCP1; therefore, this haplotype must have originated before the last common ancestor of golden jackals and other wolf-like canids >2 million years ago (Ma)^10^. Although the 16-kb HCP1-exon 2/3/4 segment haplotype could have originated on a branch leading to the core wolf-like canids, it would have had to persist via incomplete lineage sorting and absence of recombination for >2 million years and through three speciation events (Supplementary Information). A more likely scenario is that HCP1 represents a ghost lineage from an extinct canid (Figs. 4d and 5b) that was introduced by hybridization with grey wolves during the Pleistocene (below), as has been suggested for an ancestor of the grey wolf and coyote^9^ and in high-altitude Tibetan and Himalayan wolves^11^.

**Extended Data Fig. 6:**
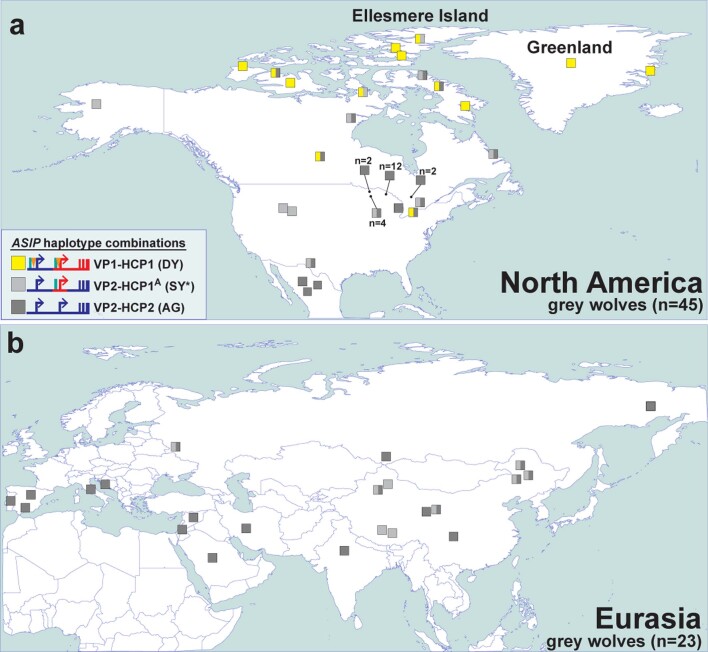
The distribution of *ASIP* haplotypes in modern grey wolves. Modern grey wolves (squares) from (**a**) North America (n = 45) or (**b**) Eurasia (n = 23) were genotyped for 5 structural variants and 6 SNVs using whole genome sequencing data. Wolves are coloured by inferred VP and HCP haplotypes, as indicated in the figure legend and in Supplementary Table 11. The asterisk indicates an SY-like haplotype without the HCP1 insertion.

**Extended Data Fig. 7:**
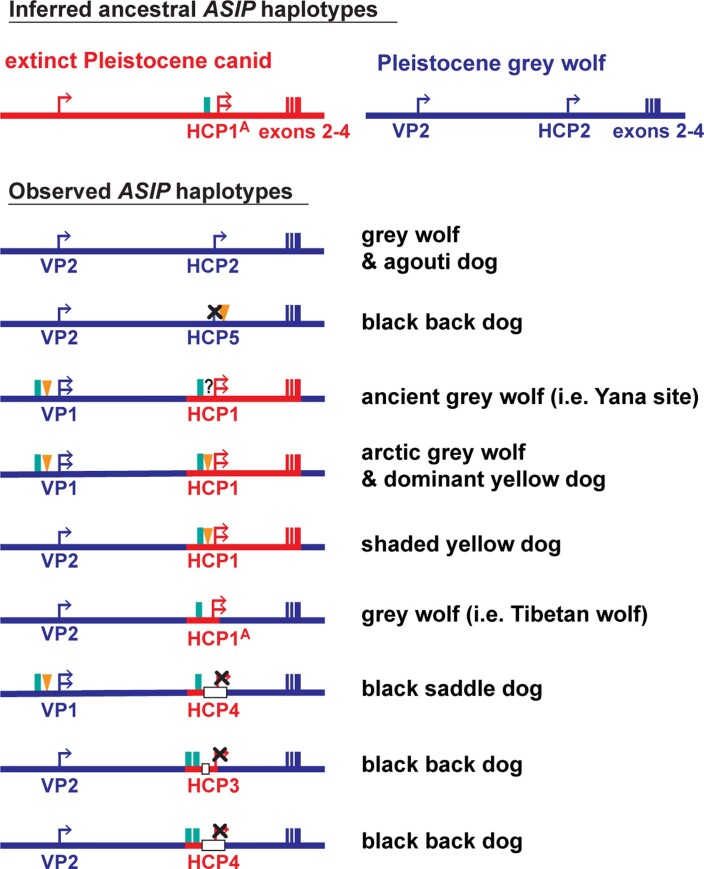
Evolutionary diversification of *ASIP* haplotypes observed in grey wolves and dogs. The colour (red or blue) of *ASIP* haplotype segments indicates ancestral species of origin, inferred from phylogenetic analysis (Fig. 4b, Extended Data Figs. 3, 4). Relevant structural variants near the ventral (VP) and hair cycle (HCP) promoters are depicted as yellow triangles (polynucleotide expansions), green bars (SINE insertions) and white bars (deletions). Modified promoter activity is indicated by an X mark (no activity) or an additional arrow (elevated expression), based on RNA-seq (Extended Data Fig. 1) and/or inference from coat colour (Figs. 1, 2c, 4c).

We expanded our analysis of VP and HCP haplotypes to a total of 45 North American and 23 Eurasian wolves. The VP1-HCP1 haplotype combination is found mostly in the North American Arctic in a distribution parallel to that of white coat colour (Extended Data Fig. 6a)^12^ and is not observed in Eurasia. We also identified an ancestral HCP1 haplotype variant, referred to hereafter as HCP1^A^, that does not extend to exons 2/3/4 and lacks the 24-bp insertion found in Arctic grey wolves and dominant yellow dogs (Fig. 4d and Extended Data Fig. 7). A haplotype combination similar to shaded yellow, VP2-HCP1^A^, was observed in seven light-coloured wolves from Tibet or Inner Mongolia, representative of a distinct, high-altitude grey wolf population that is notably lighter than other Eurasian populations (Fig. 4d and Extended Data Fig. 6b)^13^.

Additional insight into the demographic history of these haplotypes emerges from the analysis of ancient dog (*n* = 5) and grey wolf (*n* = 2) whole genome sequencing (WGS) data, dated 4–35 thousand years ago (ka) (Supplementary Information and Supplementary Table 12), in which both forms of the VP (VP1 and VP2) and four forms of the HCP (HCP1^A^, HCP1, HCP2 and HCP4) were observed in various combinations (Fig. 5a and Extended Data Fig. 7). Ancient wolves from the Lake Taimyr and Yana River areas of Arctic Siberia had at least one HCP1 haplotype, while ancient dogs from central Europe, Ireland and Siberia carried HCP1^A^, HCP1 and HCP4, respectively (Supplementary Table 12). Thus, diversity in *ASIP* regulatory sequences responsible for colour variation today was apparent by 35 ka in ancient wolves and by 9.5 ka in ancient dogs.

Together with our phylogenetic results, comparative analysis of wolf and dog *ASIP* haplotypes suggests an evolutionary history in which multiple derivative haplotypes and associated colour patterns arose by recombination and mutation from two ancestral configurations corresponding to a white wolf (VP1-HCP1) and a grey wolf (VP2-HCP2), both present in the late Pleistocene (Fig. 5a and Extended Data Fig. 7). The distribution of derivative haplotypes explains colour pattern diversity not only in dogs but also in modern wolf populations across the Holarctic, including white wolves in the North American Arctic (VP1-HCP1) and yellow wolves in the Tibetan highlands (VP2-HCP1^A^) and is consistent with natural selection for light coat colour. A likely timeline for the origin of modules driving high levels of *ASIP* expression is depicted in Fig. 5b and indicates a dual origin. The HCP1 haplotype represents introgression into Pleistocene grey wolves from an extinct canid lineage that diverged from grey wolves >2 Ma. This introgression as well as the mutation from VP2 to VP1 occurred before 33.5 ka, on the basis of direct observation from an ancient wolf sample (Fig. 5a).

## Discussion

A relationship between *ASIP* and dog colour pattern was recognized more than a century ago by Sewall Wright^14^ and explored in depth by the work of C. C. Little in the decades that followed^15^. Previous studies have reported molecular variation in or around the *ASIP* region associated with some dog colour patterns, including a 16-bp non-coding duplication associated with black saddle^16^, a SINE insertion associated with black back and black saddle^16^ and missense variants A82S and R83H associated with dominant yellow^17^ (Fig. 2a and Extended Data Fig. 2). As shown here, availability of a broader dataset indicates that these previously reported associations represent linkage disequilibrium and/or breed structure rather than causal variation (Supplementary Table 7). Instead, our WGS-based comprehensive annotation of the region, together with RNA expression data, reveals a series of structural variants that define distinct haplotypes for each of two promoters that, in combination, explain five different pattern types (Fig. 2c).

In dogs, the key differences between VP1 and VP2 are a SINE element and a small insertion; similarly, the key differences between HCP1 and HCP2 are multiple SINE elements and a small insertion (Fig. 2b). In each case, we do not yet know if the transcriptional differences (VP1 > VP2 and HCP1 > HCP2) are caused by the SINE element, the small insertion or both. We note, however, that modularity of *ASIP* regulatory variation is a general theme in vertebrates, with non-coding changes driving adaptation in natural populations of deer mice^3^, mountain hares^18^, snowshoe hares^8^ and several species of parulid warblers^19–22^. Likewise, artificial selection in goats^23^, domestic rabbits^24,25^ and laboratory mice^26^ is associated with structural variation in *ASIP* regulatory regions that may lead to acquisition of promoters that modulate region-specific expression of *ASIP*.

*ASIP* colour pattern diversification was probably an early event during dog domestication, since our analysis of ancient DNA data reveals several different VP and HCP haplotypes in Eurasia by 4.8 ka. This is consistent with the wide distribution of dominant yellow across modern dog breeds from diverse locations, as well as the dingo (Supplementary Table 9), a feral domesticate, frequently dominant yellow, introduced to Australia at least 3.5 ka (ref. ^27^). Of particular interest is the Zhokov island dog from Siberia^28,29^. On the basis of a haplotype combination of VP2-HCP4, this sled dog that lived 9.5 ka exhibited a black back colour pattern, allowing it to be easily distinguished from white-coloured wolves in an Arctic environment.

In wolves, natural selection for VP1 and HCP1 are a likely consequence of Pleistocene adaptation to Arctic environments and genetic exchange in glacial refugia, driven by canid and megafaunal dispersal during interglacial periods. Modern grey wolves are thought to have arisen from a single source ~25 ka close to the last glacial maximum^30,31^; during the North American glacial retreat that followed, the VP1-HCP1 haplotype combination was selected for in today’s white-coloured Arctic wolves. Our results show how introgression, demographic history and the genetic legacy of extinct canids played key roles in shaping diversity in dogs and modern grey wolves.

## Methods

### Ethics statement

All animal experiments were done in accordance with the local regulations. Experiments were approved by the “Cantonal Committee For Animal Experiments” (Canton of Bern; permits 48/13, 75/16 and 71/19).

### Skin biopsies and total RNA extraction

Skin biopsies (6-mm punch) were recovered from three dogs (black back Miniature Pinscher and dominant yellow Border Terrier and Irish Terrier) at necropsy and/or surgery for reasons unrelated to this study. Biopsies were recovered from the ventral abdomen and dorsal thorax and are not matched for age or hair growth cycle. The biopsies were immediately put in RNAlater (Qiagen) for at least 24 h and then frozen at –20 °C. Before RNA extraction, the skin biopsies were homogenized mechanically with the TissueLyser II device from Qiagen. Total RNA was extracted from the homogenized tissue using the RNeasy Fibrous Tissue Mini Kit (Qiagen) according to the manufacturer’s instructions. RNA quality was assessed with a Fragment Analyzer (Agilent) and the concentration was measured using a Qubit Fluorometer (ThermoFisher Scientific).

### Whole transcriptome sequencing (RNA-seq)

From each sample, 1 μg of high-quality total RNA (RNA integrity number > 9) was used for library preparation with the Illumina TruSeq Stranded mRNA kit. The libraries were individually barcoded and pooled and sequenced on an S1 flow cell with 2 × 50 bp paired-end sequencing using an Illumina NovaSeq 6000 instrument. On average, 31.5 million paired-end reads per sample were collected. One publicly available Beagle sample was used (SRX1884098). All accession numbers and descriptive read statistics are given in Supplementary Table 8. All reads that passed quality control were mapped to the CanFam3.1 reference genome assembly using STAR aligner (v.2.6.0c)^34^.

### Transcript coordinates

The STAR-aligned bam files were visualized in the integrated genomics viewer (IGV) browser^35^. Three different alternate untranslated first exons with splice junctions to the coding exons of *ASIP* were defined on the basis of the visualizations of the read alignments in IGV on the basis of the RNA-seq data just described. These exact transcripts have not been documented in the National Center for Biotechnology Information (NCBI); however, the three transcripts of NCBI annotation release 105 are virtually identical except for minor differences regarding the length of the 5′ untranslated regions (XM_014106843.2, transcription start sire (TSS) 22 nucelotides upstream compared to our annotation; NM_001007263.1, VP1-TSS 98 bp downstream of our annotation; XM_022408819.1 HCP-TSS 36 bp downstream of our annotation). Our visually curated gene models are given in Supplementary Table 2.

### Identification of genomic variants

WGS data from 71 dogs and six wolves were used for variant discovery (Supplementary Table 3). They included 15 agouti dogs and wolves, 25 black back dogs, 11 black saddle dogs, 14 dominant yellow dogs, 11 shaded yellow dogs and one white wolf. The genomes were either publicly available or sequenced as part of related projects in our group^36^. SNVs and small indels were called as described^36^. The IGV software^35^ was used for visual inspection of the three promoter regions based on the transcripts identified in the RNA-seq data. Structural variants were identified and association with coat colour phenotypes was verified by visual inspection in IGV. The pattern of copy number variation at the third promoter did not associate with the coat patterns as defined in Fig. 1.

### DNA samples for Sanger sequencing and genotyping

Samples for variant discovery included two dogs from each colour phenotype and are designated with asterisks in Supplementary Table 3. Samples from dogs listed in Supplementary Table 4 were used for genotyping. The coat colour phenotype of all animals (Supplementary Tables 3 and 4) was assigned on the basis of breed-specific coat colour standards or photographs or owner reporting. Genomic DNA was isolated from EDTA blood samples using the Maxwell RSC Whole Blood DNA kit (Promega).

### Sequencing of promoter regions

Sanger sequencing of PCR amplicons was carried out to validate and characterize structural variants in the promoter regions at the sequence level. All primer sequences and polymerases used are listed in Supplementary Table 5. PCR products amplified using LA Taq polymerase (Takara) or Multiplex PCR Kit (Qiagen) were directly sequenced on an ABI 3730 capillary sequencer after treatment with exonuclease I and shrimp alkaline phosphatase. Sequence data were analysed with Sequencher 5.1 (GeneCodes). Interspersed repeat insertions were classified with the RepeatMasker program^37^. Multiple copies of SINE elements from the same and different families were resolved this way. The CanFam3.1 reference genome assembly is derived from the Boxer Tasha, a dominant yellow dog, and represents a DY haplotype, VP1-HCP1, of the *ASIP* gene. Descriptions of the promoter variants and Genbank accession numbers for HCP2–5 are in Supplementary Table 1. Supplementary Table 1 lists the seven combinations of VP and HCP regulatory modules observed in dogs. As HCP3, 4 and 5 all represent functionally equivalent loss-of-function alleles, the seven listed combinations correspond to only five distinct phenotypes.

### Genotyping assays

Five PCR assays (ventral promoter assays 1 and 2 and hair cycle promoter assays 1, 2 and 3) were required to unambiguously determine the VP and HCP haplotypes (Supplementary Table 5). The previously reported SINE insertion^32^ was genotyped by fragment size analysis on an ABI 3730 capillary sequencer and analysed with the GeneMapper 4.0 software (Applied Biosystems). The previously reported *ASIP* coding variants^17^ were genotyped by Sanger sequencing of PCR products. The previously reported *RALY* intronic duplication^16^ was genotyped by size differentiation of PCR products on a Fragment Analyzer (Agilent). Another primer pair was used for the amplification of the entire HCP (Supplementary Table 5). Genotyping results for all samples are shown in Supplementary Table 4. There is a perfect genotype–phenotype association in 352 dogs (Table 1 and Supplementary Table 7). In the remaining 14 dogs, the presence of a eumelanistic mask prevented the reliable phenotypic differentiation of dominant yellow and shaded yellow dogs. Breeds and the different promoter haplotype combinations identified within each breed are indicated in Supplementary Table 6. In a few dogs that were heterozygous at both VP and HCP, the phasing of the VP and HCP haplotype combinations was performed on the basis of haplotype frequency within the same breed as noted. A family of Chinooks was used to determine the segregation of extended haplotypes and the phenotypic equivalency of HCP3 and 5 (Fig. 3). Summaries of genotyping results and exclusion of previously associated variants are shown in Table 1 and Supplementary Table 7. Supplementary Table 7 lists the genotype–phenotype association in aggregated form; it also contains the genotypes for variants that were previously reported to be associated with pattern phenotypes^16,17,32^.

### Comparison of promoter haplotype effects on transcripts

Transcript data were generated from a second set of samples. Sample descriptions and colours are shown in Supplementary Table 8 for all RNA experiments. Skin samples were collected from a male Swedish Elkhound (agouti), female German Pinscher (dominant yellow) and male Rottweiler (black back) after euthanasia that was conducted due to behavioural or health problems not related to skin. Samples were collected in RNAlater Stabilization Solution and stored at –80 °C. RNA was extracted using the RNeasy Fibrous Tissue Mini Kit (Qiagen) according to the manufacturer’s instructions. Integrity of RNA was evaluated with Agilent 2100 Bioanalyzer or TapeStation system (Agilent) and concentration measured with DeNovix DS-11 Spectrophotometer (DeNovix Inc.). The libraries for STRT (single cell reverse tagged) RNA-seq were prepared using the STRT method with unique molecular identifiers^38^ and modifications including longer unique molecular identifiers of 8 bp, addition of spike-in ERCC control RNA for normalization of expression and the Globin lock method^39^ with LNA-primers for the canine alpha- and beta-globin genes. The libraries were sequenced with an Illumina NextSeq 500. Reads were mapped to the CanFam3.1 genome build using HISAT1 mapper v.2.1.0 (ref. ^40^).

The alignment-free quantification method Kallisto (v.0.46.0)^41^ was used to estimate the abundance and quantified as transcripts per million mapped reads (TPM) data on the basis of an index built from CanFam3.1 Ensembl transcriptome (release 99). The curated *ASIP* transcript isoform models based on alignment visualizations in the IGV browser^35^ were also included in the transcriptome. Results based on genotype of the promoter haplotypes are displayed in Extended Data Fig. 1 as TPM.

### Haplotype construction

Haplotypes were constructed from two publicly available VCF files PRJEB32865 and PRJNA448733. The VCFs for selected dogs were merged using BCFtools merge tool (http://samtools.github.io/bcftools/) with the parameter --missing-to-ref, which assumed genotypes at missing sites are homozygous reference type 0/0. Only dogs homozygous for *ASIP* haplotypes (VP, HCP and coding exons) were used to visualize haplotypes (Supplementary Table 3). SNVs that had 100% call rate in these samples were colour coded and displayed relative to the genome assembly and previously associated variants (Extended Data Fig. 2).

### *ASIP* phylogenetic analysis in canids

Illumina whole genome sequence for 36 canids, including seven extant species and the dog, were downloaded from the NCBI short read archive as aligned (bam format) or unaligned (fastq format reads (Supplementary Table 9)). Fastq data were aligned to the dog genome (CanFam3.1) using BWA (v.0.7.17)^42^ after trimming with Trim Galore (v.0.6.4). SNVs within a 110-kb interval (chr24: 23,300,000–23,410,000), which includes the *ASIP* transcriptional unit and regulatory sequences, were identified with Platypus (v.0.8.1)^43^ and filtered with VCFtools (v.0.1.15)^44^ to include 2,008 biallelic SNVs. Phasing was inferred with BEAGLE (v.4.1)^45^.

For phylogenetic analysis, the *ASIP* interval was partitioned in two regions, on the basis of dog SNV density (Fig. 4a) and *ASIP* gene structure: a 48-kb region including the ventral first exon, extending to but excluding the hair cycle first exon (chr24:23,330,000–23,378,000) and a 16-kb region including the hair cycle first exon, extending to and including *ASIP* coding exons 2–4 (chr24: 23,378,001–23,394,000). Consensus sequences of equal length were constructed for each inferred canid haplotype using BCFtools (v.1.9). Phylogenies were inferred using maximum likelihood method and Tamura–Nei model with 250 bootstrap replications, implemented in MEGAX^46,47^ and including 34 canids (Fig. 4b and Extended Data Figs. 3 and 4). For 34 of 36 individuals, consensus haplotype pairs were adjacent to each other or, in the case of a few wolf/dog haplotypes, were positioned in neighbouring branches with weak bootstrap support. The exceptions were the African golden wolf, a species derived by recent hybridization of the grey wolf and Ethiopian wolf^9^ and an eastern grey wolf from the Great Lakes region, which was also reported to have recent admixture with the coyote^48^. The African golden wolf and the eastern grey wolf were removed from the alignments and a single haplotype for each individual was selected arbitrarily for tree building and display.

### Haplotype analysis of *ASIP* locus in ancient dogs and wolves

WGS data from several recent studies^9,13,29,49–53^, including five ancient dogs, two ancient grey wolves and 68 modern grey wolves (Supplementary Table 12) were downloaded as aligned (bam format) or unaligned (fastq format) reads. Fastq data were aligned to the dog genome (CanFam3.1) using BWA-MEM (v.0.7.17)^42^, after trimming with Trim Galore (v.0.6.4). Coverage depth for each sample ranged from 1 to 78× (Supplementary Table 12). Genotypes at five structural variants and six SNVs were determined by visual inspection using the IGV browser (Supplementary Tables 1 and 12). Variants in or near the ventral promoter (*n* = 2), the hair cycle promoter (*n* = 6) and the coding exons (*n* = 3) distinguished ventral and hair cycle promoter haplotypes (Supplementary Table 12). SNV genotypes were determined by allele counts; structural variants were genotyped by split reads at breakpoint junctions. The base maps used for plotting the geographic distribution of haplotypes (Fig. 5 and Supplementary Fig. 6) were generated in R (v.4.0.3) with ‘maps’ and ‘ggplot2’ packages.

For 67 of 75 wolves (or ancient dogs), the phase of ventral and hair cycle promoter haplotypes was unambiguous. Seven wolves and one ancient dog were heterozygous with respect to both the ventral and hair cycle promoter haplotypes and, for these samples, haplotype phase was inferred on the basis of the linkage disequilibrium in the 67 unambiguous individuals.

### Reporting Summary

Further information on research design is available in the Nature Research Reporting Summary linked to this article.

## Supplementary information

Supplementary Information Supplementary text and references.

Reporting Summary

Peer Review Information

Supplementary Table Excel file with 12 tabs, one for each table. Each table has a title.

## Data Availability

All data generated or analysed during this study are included in this published article and its Supplementary Information. GenBank accession numbers for promoter sequence variants are MT319114.1, MT319115.1, MT319116.1 and MT319117.1.
