# Supplementary material for: Dog colour patterns explained by modular promoters of ancient canid origin
Source: Nat Ecol Evol. 2021 Aug 12;5(10):1415–23. doi: 10.1038/s41559-021-01524-x (PMC8484016; doi:10.1038/s41559-021-01524-x)
Supplement: Supplementary file 2 — Reporting Summary [file 41559_2021_1524_MOESM2_ESM.pdf]

nature research

Corresponding author(s): Danika Bannasch

Last updated by author(s): Apr 22, 2021

Reporting Summary

Nature Research wishes to improve the reproducibility of the work that we publish. This form provides structure for consistency and transparency in reporting. For further information on Nature Research policies, see our Editorial Policies and the Editorial Policy Checklist.

Statistics

For all statistical analyses, confirm that the following items are present in the figure legend, table legend, main text, or Methods section.

n/a	Confirmed	
☐	☒	The exact sample size (*n*) for each experimental group/condition, given as a discrete number and unit of measurement
☐	☒	A statement on whether measurements were taken from distinct samples or whether the same sample was measured repeatedly
☐	☒	The statistical test(s) used AND whether they are one- or two-sided<br>*Only common tests should be described solely by name; describe more complex techniques in the Methods section.*
☒	☐	A description of all covariates tested
☒	☐	A description of any assumptions or corrections, such as tests of normality and adjustment for multiple comparisons
☒	☐	A full description of the statistical parameters including central tendency (e.g. means) or other basic estimates (e.g. regression coefficient) AND variation (e.g. standard deviation) or associated estimates of uncertainty (e.g. confidence intervals)
☒	☐	For null hypothesis testing, the test statistic (e.g. *F*, *t*, *r*) with confidence intervals, effect sizes, degrees of freedom and *P* value noted<br>*Give P values as exact values whenever suitable.*
☒	☐	For Bayesian analysis, information on the choice of priors and Markov chain Monte Carlo settings
☒	☐	For hierarchical and complex designs, identification of the appropriate level for tests and full reporting of outcomes
☒	☐	Estimates of effect sizes (e.g. Cohen's *d*, Pearson's *r*), indicating how they were calculated

Our web collection on statistics for biologists contains articles on many of the points above.

Software and code

Policy information about availability of computer code

Data collection	No software was used for data collection.
Data analysis	All software used was referenced in the methods section and the version used was provided.

For manuscripts utilizing custom algorithms or software that are central to the research but not yet described in published literature, software must be made available to editors and reviewers. We strongly encourage code deposition in a community repository (e.g. GitHub). See the Nature Research guidelines for submitting code & software for further information.

Data

Policy information about availability of data

All manuscripts must include a data availability statement. This statement should provide the following information, where applicable:

- Accession codes, unique identifiers, or web links for publicly available datasets
- A list of figures that have associated raw data
- A description of any restrictions on data availability

All data generated or analyzed during this study are included in this published article (and its supplementary information files). GenBank accession numbers for promoter sequence variants are MT319114.1, MT319115.1, MT319116.1, MT319117.1.

Field-specific reporting

Please select the one below that is the best fit for your research. If you are not sure, read the appropriate sections before making your selection.

☒ Life sciences ☐ Behavioural & social sciences ☐ Ecological, evolutionary & environmental sciences

For a reference copy of the document with all sections, see nature.com/documents/nr-reporting-summary-flat.pdf

Life sciences study design

All studies must disclose on these points even when the disclosure is negative.

Sample size	Dogs and and breeds were selected for genotyping based on segregation of coat colors within and between breeds. Some breeds were included if they had been reported to have coat colors inconsistent with previously identified variants. Publicly available whole genome sequenced samples of dogs, wild canids and ancient samples were used. For phylogenetic analysis, whole genome sequence from at least one representative for each extant canid species was included, based on data availability.
Data exclusions	All data exclusion was noted. Rational for excluding individuals samples in phylogenetic analysis are addressed in the methods section and based on evidence in prior studies.
Replication	Phylogenies were inferred using Maximum Likelihood (Tamura-Nei model) with 250 bootstrap replications.
Randomization	Dogs are grouped by color pattern as outlined in Figure 1 and described in the main text.
Blinding	Classification of dogs by color pattern was done prior to genetic and genomic analyses, based on standard practices for genetic association studies.

Reporting for specific materials, systems and methods

We require information from authors about some types of materials, experimental systems and methods used in many studies. Here, indicate whether each material, system or method listed is relevant to your study. If you are not sure if a list item applies to your research, read the appropriate section before selecting a response.

Materials & experimental systems

n/a	Involved in the study	
☒	☐	Antibodies
☒	☐	Eukaryotic cell lines
☒	☐	Palaeontology and archaeology
☐	☒	Animals and other organisms
☒	☐	Human research participants
☒	☐	Clinical data
☒	☐	Dual use research of concern

Methods

n/a	Involved in the study	
☒	☐	ChIP-seq
☒	☐	Flow cytometry
☒	☐	MRI-based neuroimaging

Animals and other organisms

Policy information about studies involving animals; ARRIVE guidelines recommended for reporting animal research

Laboratory animals	The study does not involve the use of laboratory animals.
Wild animals	Genomic data from natural populations of wild animals was obtained from prior studies.
Field-collected samples	DNA samples from owned pet dogs.
Ethics oversight	All animal experiments were done in accordance with the local regulations. Experiments were approved by the “Cantonal Committee For Animal Experiments” (Canton of Bern; permits 48/13, 75/16 and 71/19).

Note that full information on the approval of the study protocol must also be provided in the manuscript.
