# Supplementary material for: Dog colour patterns explained by modular promoters of ancient canid origin
Source: Nat Ecol Evol. 2021 Aug 12;5(10):1415–23. doi: 10.1038/s41559-021-01524-x (PMC8484016; doi:10.1038/s41559-021-01524-x)
Supplement: Supplementary file 3 — Peer Review Information [file 41559_2021_1524_MOESM3_ESM.pdf]

natureresearch

Peer Review Information

Journal: Nature Ecology & Evolution
Manuscript Title: Dog color patterns explained by modular promoters of ancient canid origin
Corresponding author name(s): Danika L. Bannasch

Editorial Notes:

Reviewer Comments & Decisions:

Decision Letter, initial version:

15th March 2021

*Please ensure you delete the link to your author homepage in this e-mail if you wish to forward it to your co-authors.

Dear Danika,

Your manuscript entitled "Dog color patterns explained by modular promoters of ancient canid origin" has now been seen by 3 reviewers, whose comments are attached. The reviewers have raised a number of concerns which will need to be addressed before we can offer publication in Nature Ecology & Evolution. We will therefore need to see your responses to the criticisms raised and to some editorial concerns, along with a revised manuscript, before we can reach a final decision regarding publication.

We therefore invite you to revise your manuscript taking into account all reviewer and editor comments. Please highlight all changes in the manuscript text file in Microsoft Word format.

We are committed to providing a fair and constructive peer-review process. Do not hesitate to contact us if there are specific requests from the reviewers that you believe are technically impossible or unlikely to yield a meaningful outcome.

When revising your manuscript:

* Include a “Response to reviewers” document detailing, point-by-point, how you addressed each reviewer comment. If no action was taken to address a point, you must provide a compelling argument. This response will be sent back to the reviewers along with the revised manuscript.

* If you have not done so already please begin to revise your manuscript so that it conforms to our Article format instructions at http://www.nature.com/natecolevol/info/final-submission. Refer also to any guidelines provided in this letter.

* Include a revised version of any required reporting checklist. It will be available to referees (and, potentially, statisticians) to aid in their evaluation if the manuscript goes back for peer review. A revised checklist is essential for re-review of the paper.

Please use the link below to submit your revised manuscript and related files:

[REDACTED]

Note: This URL links to your confidential home page and associated information about manuscripts you may have submitted, or that you are reviewing for us. If you wish to forward this email to co-authors, please delete the link to your homepage.

We hope to receive your revised manuscript within four to eight weeks. If you cannot send it within this time, please let us know. We will be happy to consider your revision so long as nothing similar has been accepted for publication at Nature Ecology & Evolution or published elsewhere.

Nature Ecology & Evolution is committed to improving transparency in authorship. As part of our efforts in this direction, we are now requesting that all authors identified as 'corresponding author' on published papers create and link their Open Researcher and Contributor Identifier (ORCID) with their account on the Manuscript Tracking System (MTS), prior to acceptance. ORCID helps the scientific community achieve unambiguous attribution of all scholarly contributions. You can create and link your ORCID from the home page of the MTS by clicking on 'Modify my Springer Nature account'. For more information please visit please visit www.springernature.com/orcid.

Please do not hesitate to contact me if you have any questions or would like to discuss these revisions further.

We look forward to seeing the revised manuscript and thank you for the opportunity to review your work.

[REDACTED]

Reviewer expertise:

Reviewer #1: genetics of colour variation in mammals

Reviewer #2: evo-devo of vertebrate pigmentation

Reviewer #3: dog genomics

Reviewers' comments:

Reviewer #1 (Remarks to the Author):

The agouti (ASIP) protein regulates pigmentation in vertebrates by acting on the MC1R receptor.

Mutation or variation in the ASIP gene has been associated with pigment pattern phenotypes. The gene structure and regulation is best understood is in mice where two promoters drive the same protein coding sequence either on the ventral part of the body or across the whole body in a hair-cycle coordinated fashion. Mutations in one or the other promoter results in characteristic patterns. Based on variant coat pigmentation patterns, it has been understood for some time that dogs probably have the same two ASIP promoters and the patterns can be explained by mutation of either of them.

This manuscript is a comprehensive and thorough analysis of the promoter structure of ASIP in dogs. They characterize 7 different combinations of promoter haplotypes, made up of 2 forms of the ventral promoter combined with 5 forms of hair cycle promoter which give rise to 5 phenotypes. They demonstrate by RNA-seq that the ventral promoter haplotype associated with more yellow coat has a ~6x higher expression than the agouti-associated one, and the hair-cycle promoters associated with black dorsums are inactive.

Most significantly they find that the yellow-associated haplotype is also seen in white arctic grey wolves. When compared to other dog species and domestic breeds the distal part of the sequence clusters in a distinct and separate clade. The proximal sequences cluster in the expected phylogeny. They suggest that this distal haplotype has arisen in a now-extinct ancestor. They extend the haplotype analysis across further modern wolves but also examine ancient dog and wolf DNA. Remarkably they find a range of haplotypes in dogs going back over 9,000 years. Furthermore they find the yellow-associated haplotype in wolves from 33,000 years ago, indicating that the white or pale wolves evolved during or before the last ice age.

This is an excellent and insightful piece of work and I have only very few minor comments:

Line 69, text says they identify 3 alternative first exons, but Figure 2 and legend, line 103 mentions 2 relevant first exons. What is the third?

The say that the VP1 promoter is expressed more widely than VP2, which fits with the phenotype of black-saddle versus black-back. The former has the yellow ventrum extend further dorsally, Figure 2). But in Extended Data Figure 1 they show VP1 expression in the dorsal thorax, which is black in the black-saddle dogs. Wouldn't we expect the dorsum to be yellow?

Reviewer #2 (Remarks to the Author):

Bannasch et al examine structural variation in the ASIP pigmentation locus and identify variants associated with different coat phenotypes in dogs. Then, performing phylogenetic analyses with wolves, suggest a possible explanation for the origin and spread of the different ASIP variants found in modern dogs.

I find this manuscript very interesting and the analysis is thorough. I have a few suggestions for improving the overall quality:

- There is substantial variation within the 5 phenotypic categories that the authors propose, implying that there are many other factors that participate in establishing the patterns seen in each group. While ASIP is clearly a gene of major effect, it is important that the authors acknowledge this fact explicitly

- It is important to also mention more explicitly that RNAseq was performed in a subset of phenotypes and not in all of them. The description of Fig 3b should include a statement saying that expression

levels are inferred based on structural variation

- Related to the point above, the authors do not provide any direct evidence that such structural variations indeed have any relevant functional activity in the regulation of ASIP. Granted, the strong association between structural variation and phenotype is solid evidence, the authors should either either perform some sort of functional experiment (e.g., ATACseq) on a selected number of phenotypes and show that peaks map to elements near the HCP or, at the very least clarify this point and add this significant caveat to their claims.

- The results from Fig 3b and subsequent phylogenetic reconstructions suggests that the VP and HCP seem to be modular and follow different evolutionary histories. The authors provide a thorough account for the patterns seen but they should expand on this topic to include a possible explanation as to how these patterns may have come to be. For example, is the assumption that selection operated on each promoter independently. Work from the Hoekstra lab has shown that ASIP is a particularly modular gene and that selection can act on different regions to drive more granular elements of the phenotype in question. Is something similar going on here? It would be interesting to discuss these findings in this context.

Reviewer #3 (Remarks to the Author):

Colour variation in mammals, and across the vertebrate tree, is a long standing area of interest for geneticists. Rightly or wrongly, external colour patterns have been used to infer domestic individuals from their wild or feral counterparts. However, colour is not a simple phenotype. Multiple locus names and terminologies are used to describe the same pattern, and the final observed phenotype is the product of multiple interacting genes, e.g. MC1R, ASIP, CBD103 etc.

Here, the authors focus on the canine ASIP locus, aiming to identify the alternate gene transcripts. Once these were defined, surrounding genetic variation was used to characterise the suspected regulatory regions of alternative promoters, and then tie these haplotypes to the regulation of both ventral (V) and hair cycle (HC) ASIP gene expression. This initial investigation and haplotype dissection was conducted across domestic dogs, before further analysis was conducted in wolves, canids and ancient dogs. The result is an exploration of extended V and HC haplotypes across canids, suggesting ASIP colour patterns in dogs occurred during domestication, and are not based on a pool of standing regulatory variants present in the most recent common ancestor pool.

I commend the authors on the harmonisation of ASIP pattern names, and their ASIP RNA and DNA sequencing efforts. The use of existing WGS data extended their results past the avenue of domestic interest and into canid colour evolution. Their results will be of interest to many readers, but some revision is required.

Why did the authors exclude the a third canine alternate promoter in favour of the orthologous to mouse ventral (VP) and hair cycle promoters (HCP). Given the authors then go on to state it VP and HCP that drive colour, what role is hypothesised for the third promoter transcript? It seems to be full length. There are examples of other mammals with more than two alternative ASIP promoters [https://doi.org/10.1016/j.ygeno.2009.11.003].

As the authors note in the description of figure 2, MC1R can interfere with the characterisation of ASIP patterns. It would be better if the interplay between suspected colour genes (e.g. ASIP, MCIR, RALY), and their mentioned resultant colour morphs including the “mask” or “dark” phenotypes, were described briefly in the introductory text. This would help to reader to follow stated limitations. If the reader was not familiar with RALY, it’s sudden inclusion on line 369 would come as a surprise. It does

also lead the reader open to wondering why MC1R alleles were typed in some individuals (with WGS) and not others. Perhaps the authors could comment.

The authors purport to define ASIP regulatory modules, but no hypothesis is given for the loss of function action for HCP3 and 5. It seems that HCP cassette would lend itself to this discussion, whether it be structural rearrangement, attraction of methylation etc. If the authors cannot rule out LOF coding variants in phase with these 2 haplotypes, this should be noted.

From figure 2, it is also not clear if the ASIP regulatory modules contain the known ASIP SINE element [https://doi.org/10.1093/jhered/esr042], or which one it is. The location of additional ASIP and RALY variants (mentioned in Supplementary Table 7) is also slightly cryptic. Perhaps the authors could be inspired by Figure 2 of Fontanesia et al., [https://doi.org/10.1016/j.ygeno.2009.11.003] and their depiction of known and novel locus transcripts and variants. There, for the rabbit, the relative positions of the key transcripts and associated polymorphisms are stated. This information is not easily available in the current version of the manuscript and required flicking between tables or looking at reference texts.

The discussion of V an HC promoter haplotypes can become confusing to read, especially as different pots of individuals are added to the analyses, and new haplotypes are uncovered in wolves (HCP1 becomes HCP1a an1b). However, the methods used, and the subsequent interpretation of phylogenies and evolutionary models is fairly clear and not overstated. Some cleaning up of text in the manuscript and supplementary text would help.

Of note, dingos, like domestic dogs, are not a single colour [https://doi.org/10.1111/jzo.12875]. This existing diversity is consistent with the author's statement of lines 198-199 for the timing of colour variation (depending on accepted dingo age 3,500-8,000 ybp), but lines 233-234 may misrepresent the dingo group as only having the dominant yellow colour.

Detailed notes.
Figure 2a. Please add genomic coordinates for the SINE and LINE elements, or if not possible, please indicate their size and orientation. This may make Fig 2a too busy, but this information should be reported in some fashion in the manuscript and then linked back to this figure. This could help the reader understand why HCP3 and 5, with intact exon 2 are loss of function haplotypes. Are the duplicate copies of SINE C2A1_CF full length? This could impact the regulatory potential of the element.
Can the authors place the known SINE element from Dreger et al. 2011 in the context of their figure 2a? This element is genotyped in the paper but its relationship to the newly reported SINEs is not clear.

Figure 2b could be misleading. At first glance it appears that the semi-quantitative expression was measured in the number of individuals (N) under the dog illustrations. This is not the case. Rather these numbers seem to reflect the 352 individuals genotyped from supplementary Table 5. This is important, as there are very few data points to support the transcription analysis (only one data point for VP1 in Extended Data Fig1). The authors report Extended Data Fig1 in their figure description, but difference between RNA seq results and genotyping could be clearer.

The authors state that HCP3-5 are loss of function haplotypes, as evidenced by the lack of transcripts identified from the RNA-seq. Was it possible to extract DNA from the tissues samples at the same time as RNA to confirm no other LOF variants in the transcripts, and so assure the reader that the LOF is from the regulatory module?

Why did the authors discount the third ASIP transcription start site in their analysis? It is illustrated in

Extended Data Figure 2, but is not shown in Figs 2 or 3 in the main text. Do the authors not believe it is a true, or translated transcript? Could it have had the potential to influence the colours described here?

Figure 3. Is the Tibetan wolf (Supplementary Table 10) part of fig 3b? Extended data figures 4 and 5 show it as being part of this analysis. If that is so, is this analysis based solely on the individuals in Supplementary Table 9 (line 123), or are individuals in Supplementary Table 10 also included?

Line 126. Do you mean Fig 3b and 3c here?

Line 128. Which polymorphic site is variable between grey wolves? Extended Data Table 2 does to report that information. In my copy of Fig2 some genotypes appear to be missing (white), but I assume the variant being referred to is closer to the 23.33 Mb end.

Extended Data Table 2. What is a Yana wolf?

Supplementary Table 1. Shaded yellow and dominant yellow have the same accession number but represent different haplotypes. Is one of these incorrect?
Authors use HGVS nomenclature in other supplementary tables, but not this table. Please be consistent and use HGVS throughout.

Lines 76-77, 337-339 and Supplementary Table 2. 77 samples are described in the text and reported in the table, but the designations do not match. Please fix. Under “color” in the table, 5 wolves are described as “wolf”, not a coat colour. These “n.d.” individuals seem to be merged with the agouti individuals in the text description.

Lines 337-339. How many genomic variants were detected from the WGS data of 77 dogs and wolves? How were these dissected for segregation pattern to coat colour? Were only promoter regions considered for colour outcome, or was the 30kb gene space considered? Were variants phased?

Lines 311-328. Skin biopsies and RNA sequencing. Six skin biopsies are described, two per individual. Was each of the libraries barcoded before sequencing? If yes, can the authors report the range of reads per sample, rather than the average?

Lines 330-335. Transcript coordinates. The published sample, SRX1884098, is mentioned as being retrieved during the whole transcript sequencing phase. What were the transcript coordinates for this sample? If it was not used during the alignment phase, what was its purpose? In Supplementary Table 2, it is not noted which gene models derived from which samples. If this is known, can the authors please report the findings? How do the three new transcripts relate to the provisional RefSeq transcript, NM_001007263.1?

Lines 341-342. “...used for visual inspection of the promoter regions based on the transcripts identified in the RNA sequencing data.” Does this include the three ASIP transcripts in Supplementary Table 2, or only the two that are described in more detail throughout the manuscript?

Why is Supplementary Table 5 (line 346) mentioned before Supplementary Table 4 (line 353) in the methods? Can these numbers be swapped?

Lines 365-374 Genotyping. Variable markers from three publications are mentioned in the genotyping section, but their relationship to the newly discovered elements is not clear. Please update Supplementary Table 4 to include the HGVS location of all new targets and perhaps present these in physical location order.

Lines 412. What region was considered in the haplotype reconstruction? PRJEB32865 is not publicly available. Is this being updated? From SRA, “No public data is linked to this project.” PRJNA448733 is “... 722 genomes sequenced via WGS containing various wild canids, dingo, and domesticated dogs.” Are 722 considered at this stage?

Supplementary Table 3. How was the colour of each individual assigned? Was this in the same fashion as for Supplementary Table 5 (see text lines 347-348)?

Supplementary Tables 4/5/7. From the title description, “...Genotypes at previously used diagnostic markers (5,6,7) are also given.” Does 5,6,7 indicate references? From which reference section, there are three to chose from. Please link the references to each table, with PMIS, doi or extended identifier.

Supplementary Table 5 notes the genotyping of “Black and tan insertion: NC_006606.3:g.23365284_23365285insHQ910237; N=no insertion, I=insertion” and gives the result over two boxes (assume one for each chromosome). How is the reader to interpret the variant nomenclature? Is the full 1113bp of HQ910237 genotyped, or just the sine element? Supplementary Table 4 seems to indicate only 160bp of a SINE element. Suggest the authors reformat their description to reflect HGVS (e.g. NC_006606.3:g.23365284_23365285insHQ910237:X_Y, where X and Y are the relevant bases of HQ910237).

Supplementary Table 8 notes “ASIP mean coverage depth”, but not every sample has a value. Could the missing samples not be aligned? Seems unlikely as some missing samples were retrieved as aligned reads.

Supplementary Table 10. What is the difference between no entry in the table and n.d.? What does n.d. mean? What is “Collection site coordinates”? What is “?” in “HCP Repeat Elements”. Does SAMN14210384 have true deletions or are is this an issue to do with the age of the sample? If true deletion, please state break points.

Extended Data Fig 2. The figure would benefit from the annotation of genomic positions, For example, 70kb to RALY Dup. 70kb from where? What is the size of the blocks considered in the haplotype figure? How many individuals are considered in each colour class? I estimate 7 in AG, and this matches Sup Table 3, but it would help to label the figure as DY is 8, BS is 5 etc, but the haplotype blocks are very similar in size. The colour “wolf” is indicated to be used in this section from Sup Table 3, but I do not see it in the figure. What is a “more primitive breed” as described by the authors in this figure legend? Were they genotyped in this paper, or were they used in the publications referred to in this work?

In the supplementary tables but not the text, the authors discuss the incorrect segregation of commercial markers to phenotype. Seems strange to use the terms "commercial" here, but refer to the publications that identified them in the rest of the text.

Be consistent with naming. For example, Fig. 3B or Fig. 3b in the descriptions of Extended Figures 4 and 5. Fig. 2B or Fig. 2b? Both are used in the text. Is the reference canFam3.1 or CanFam3.1? Both are used in the text.
Check that correct image table is being sited. For example, Supplementary Table 2 refers to FigS2, but there is no FigS2 in the manuscript. Suspect this is for Extended Figure 2.

********************END********************

Author Rebuttal to Initial comments

Reviewer expertise:

Reviewer #1: genetics of colour variation in mammals

Reviewer #2: evo-devo of vertebrate pigmentation

Reviewer #3: dog genomics

Authors response: We thank all three reviewers for thoughtful comments that have allowed us to better present our work. At the same time we were tasked with formatting the paper specifically for Nature Ecology and Evolution. We wish to point out to the reviewers the major changes in formatting. We divided the previous Fig 2 into a revised Figure 2 and Table 1. Extended data has now been moved to supplementary except for Extended data Figure 3 which became Figure 3 in the main file. This changed the figure number for Figures 3 and 4 in the manuscript to Figures 4 and 5 respectively. Likewise in the supplemental section figures were renumbered from 3 to 7. The figures have been removed and submitted as AI files and the figure legends have been moved to the end of the main file. All changes to the text are submitted as track changes. Our responses are in italics. Line numbers refer to the line numbers from the tracked changes document set in "all mark-up mode".

Reviewers' comments:

Reviewer #1 (Remarks to the Author):

The agouti (ASIP) protein regulates pigmentation in vertebrates by acting on the MC1R receptor. Mutation or variation in the ASIP gene has been associated with pigment pattern phenotypes. The gene structure and regulation is best understood is in mice where two promoters drive the same protein coding sequence either on the ventral part of the body or across the whole body in a hair-cycle coordinated fashion. Mutations in one or the other promoter results in characteristic patterns. Based on variant coat pigmentation patterns, it has been understood for some time that dogs probably have the same two ASIP promoters and the patterns can be explained by mutation of either of them.

This manuscript is a comprehensive and thorough analysis of the promoter structure of ASIP in dogs. They characterize 7 different combinations of promoter haplotypes, made up of 2 forms of the ventral promoter combined with 5 forms of hair cycle promoter which give rise to 5 phenotypes. They demonstrate by RNA-seq that the ventral promoter haplotype associated with more yellow coat has a ~6x higher expression than the agouti-associated one, and the hair-cycle promoters associated with black dorsums are inactive.

Most significantly they find that the yellow-associated haplotype is also seen in white arctic grey wolves. When compared to other dog species and domestic breeds the distal part of the sequence clusters in a distinct and separate clade. The proximal sequences cluster in the expected phylogeny. They suggest that this distal haplotype has arisen in a now-extinct ancestor. They extend the haplotype analysis across further modern wolves but also examine ancient dog and wolf DNA. Remarkably they find a range of haplotypes in dogs going back over 9,000 years. Furthermore they find the yellow-associated haplotype in wolves from 33,000 years ago, indicating that the white or pale wolves evolved during or before the last ice age.

This is an excellent and insightful piece of work and I have only very few minor comments:

Line 69, text says they identify 3 alternative first exons, but Figure 2 and legend, line 103 mentions 2 relevant first exons. What is the third?

The third alternative first exon and promoter lie ~16 kb upstream of the ventral promoter. Transcript levels from this promoter did not vary in dorsal and ventral skin, and do not contribute to variation in the five pattern types described here. Figure 2 and the text (Beg line 67) have been modified accordingly.

The say that the VP1 promoter is expressed more widely than VP2, which fits with the phenotype of black-saddle versus black-back. The former has the yellow ventrum extend further dorsally, Figure 2). But in Extended Data Figure 1 they show VP1 expression in the dorsal thorax, which is black in the black-saddle dogs. Wouldn't we expect the dorsum to be yellow?

With regard to the reviewer's concern, the point of Supplementary Fig 1 (formerly Extended Data Figure 1) is that VP1 is expressed at higher levels than VP2, evident in both dorsal and ventral skin biopsy samples. Because our sampling protocol did not allow matching for age or hair cycle, we refer to the results as semi-quantitative. Supplementary Table 8 has also been modified to make it easier to see which samples were used. Finally, we note that the phenotype pattern descriptions are based on gross appearance rather than hair microscopy or pigment type analyses, and that black areas in, e.g. a black saddle dog, may contain small amounts of pheomelanin. These points have been clarified in the legend to Supplementary Fig 1.

Reviewer #2 (Remarks to the Author):

Bannasch et al examine structural variation in the ASIP pigmentation locus and identify variants associated with different coat phenotypes in dogs. Then, performing phylogenetic analyses with wolves, suggest a possible explanation for the origin and spread of the different ASIP variants found in modern dogs.

I find this manuscript very interesting and the analysis is thorough. I have a few suggestions for improving the overall quality:

- There is substantial variation within the 5 phenotypic categories that the authors propose, implying that there are many other factors that participate in establishing the patterns seen in each group. While ASIP is clearly a gene of major effect, it is important that the authors acknowledge this fact explicitly.

We agree, and have explained and acknowledged this point explicitly in the legend to Fig. 1.

- It is important to also mention more explicitly that RNAseq was performed in a subset of phenotypes and not in all of them. The description of Fig 3b should include a statement saying that expression levels are inferred based on structural variation

We agree with the first point and have stated this explicitly in the Results (line 66) when referring to the initial transcript definitions, methods (lines 352-356), legend to Supplementary Fig. 1 and we modified supplementary Table 8 for clarification. With regard to the second point, expression levels are inferred from a combination of RNA-seq data (from black back and/or dominant yellow dogs), structural variation, and phenotype. This is now stated explicitly in the legend to Fig. 2.

- Related to the point above, the authors do not provide any direct evidence that such structural variations indeed have any relevant functional activity in the regulation of ASIP. Granted, the strong association between structural variation and phenotype is solid evidence, the authors should either either perform some sort of functional experiment (e.g., ATACseq) on a selected number of phenotypes and show that peaks map to elements near the HCP or, at the very least clarify this point and add this significant caveat to their claims.

We agree that ATAC-seq would add additional information. However, this would require obtaining substantial amounts of material (because microdissection or scATAC-seq would be necessary) from dogs that are matched for age and hair cycle, and would therefore entail additional ethical concerns regarding live animals. We note, however, that the structural variants we ascribe to the VP and HCP lie in close proximity (< 1.5 kb) of the respective transcriptional start sites, which is now apparent in a revised

version of Fig. 2. In addition as the reviewer suggests, we have clarified this point and acknowledged the caveat in the Discussion (Line 222-223).

- The results from Fig 3b and subsequent phylogenetic reconstructions suggests that the VP and HCP seem to be modular and follow different evolutionary histories. The authors provide a thorough account for the patterns seen but they should expand on this topic to include a possible explanation as to how these patterns may have come to be. For example, is the assumption that selection operated on each promoter independently. Work from the Hoekstra lab has shown that ASIP is a particularly modular gene and that selection can act on different regions to drive more granular elements of the phenotype in question. Is something similar going on here? It would be interesting to discuss these findings in this context.

We agree with this point and have expanded the Discussion section accordingly (lines 225-229), including references to Linnen et al. as well as analogous work in other vertebrates.

Reviewer #3 (Remarks to the Author):

Colour variation in mammals, and across the vertebrate tree, is a long standing area of interest for geneticists. Rightly or wrongly, external colour patterns have been used to infer domestic individuals from their wild or feral counterparts. However, colour is not a simple phenotype. Multiple locus names and terminologies are used to describe the same pattern, and the final observed phenotype is the product of multiple interacting genes, e.g. MC1R, ASIP, CBD103 etc.

Here, the authors focus on the canine ASIP locus, aiming to identify the alternate gene transcripts. Once these were defined, surrounding genetic variation was used to characterise the suspected regulatory regions of alternative promoters, and then tie these haplotypes to the regulation of both ventral (V) and hair cycle (HC) ASIP gene expression. This initial investigation and haplotype dissection was conducted across domestic dogs, before further analysis was conducted in wolves, canids and ancient dogs. The result is an exploration of extended V and HC haplotypes across canids, suggesting ASIP colour patterns in dogs occurred during domestication, and are not based on a pool of standing regulatory variants present in the most recent common ancestor pool.

I commend the authors on the harmonisation of ASIP pattern names, and their ASIP RNA and DNA sequencing efforts. The use of existing WGS data extended their results past the avenue of domestic interest and into canid colour evolution. Their results will be of interest to many readers, but some revision is required.

Why did the authors exclude the a third canine alternate promoter in favour of the orthologous to

mouse ventral (VP) and hair cycle promoters (HCP). Given the authors then go on to state it VP and HCP that drive colour, what role is hypothesised for the third promoter transcript? It seems to be full length. There are examples of other mammals with more than two alternative ASIP promoters [https://doi.org/10.1016/j.ygeno.2009.11.003].

Unlike the VP and HCP, transcripts from the third promoter, which lies ~ 16 kb upstream from the VP, did not vary in abundance and/or structure in our dataset, and therefore we do not know the function of the third promoter, if any. This point is stated explicitly in the revised manuscript (line 76). We agree that ASIP alternative promoters and modularity of regulation is a general theme in vertebrate evolution (in rabbits, hares, deer mice, and parulid warblers) and have briefly commented on this point in the Discussion (lines 223-229).

As the authors note in the description of figure 2, MC1R can interfere with the characterisation of ASIP patterns. It would be better if the interplay between suspected colour genes (e.g. ASIP, MCIR, RALY), and their mentioned resultant colour morphs including the “mask” or “dark” phenotypes, were described briefly in the introductory text. This would help to reader to follow stated limitations. If the reader was not familiar with RALY, it’s sudden inclusion on line 369 would come as a surprise.

We agree that the reference to RALY may have been confusing. The important point is that previous work by Dreger et al. noted an association between an intronic indel in RALY with an ASIP pattern phenotype, and that our data show the association is due to linkage and/or breed structure rather than causal variation. This point is now addressed explicitly in the Supplemental Fig. 2, and the position of RALY and the intronic indel is shown in Fig. 2a. We acknowledge and agree that genes other than ASIP such as MC1R, CBD103, and various white spotting genes interact in a way that can make phenotype to genotype inferences complicated. However, because the subject of the manuscript is ASIP-associated variation and its evolutionary origin in canids, we think it is better to reserve a discussion of genetic interactions for an audience specifically interested in dog coat color genetics.

It does also lead the reader open to wondering why MC1R alleles were typed in some individuals (with WGS) and not others. Perhaps the authors could comment.

Association between the black mask phenotype and MC1R variation is not perfect, so genotyping MC1R in the expanded panel of dogs would not have led to unambiguous inferences regarding ASIP pattern types.

The authors purport to define ASIP regulatory modules, but no hypothesis is given for the loss of function action for HCP3 and 5. It seems that HCP cassette would lend itself to this discussion, whether it be structural rearrangement, attraction of methylation etc. If the authors cannot rule out LOF coding variants in phase with these 2 haplotypes, this should be noted.

We can, indeed, rule out LOF coding variants; as noted in the reply to reviewer #2, the structural variants that define the different HCP (and VP) haplotypes are located in close proximity, < 1.5 kb, to the promoter (revised Figure 2). We agree that additional data and discussion supporting our conclusion regarding LOF for HCP3, 4, and 5 would be helpful. We have modified the results to include a main figure (Fig. 3) and supplementary material to show that HCP3, 4, and 5 are non-complementing, and to note that HCP4 includes a deletion of the hair cycle transcriptional start site and non-coding exon 1 (lines 103-109).

From figure 2, it is also not clear if the ASIP regulatory modules contain the known ASIP SINE element [https://doi.org/10.1093/jhered/esr042], or which one it is. The location of additional ASIP and RALY variants (mentioned in Supplementary Table 7) is also slightly cryptic. Perhaps the authors could be inspired by Figure 2 of Fontanesia et al., [https://doi.org/10.1016/j.ygeno.2009.11.003] and their depiction of known and novel locus transcripts and variants. There, for the rabbit, the relative positions of the key transcripts and associated polymorphisms are stated. This information is not easily available in the current version of the manuscript and required flicking between tables or looking at reference texts.

We thank the reviewer for this suggestion and have used Figure 2 of the referenced work as a model to revise our Fig. 2 as well as Supplementary Fig 2, with the goal of showing more clearly the relationship between structural variants that define the VP or HCP, and those that have been previously associated with ASIP.

The discussion of V and HC promoter haplotypes can become confusing to read, especially as different pots of individuals are added to the analyses, and new haplotypes are uncovered in wolves (HCP1 becomes HCP1a an1b). However, the methods used, and the subsequent interpretation of phylogenies and evolutionary models is fairly clear and not overstated. Some cleaning up of text in the manuscript and supplementary text would help.

We have modified the text and figures in an attempt to be more clear and consistent with our discussion of promoter haplotypes in wolves. In particular, we now use the term $HCP1^A$ to refer to the ancestral version of HCP1 in wolves.

Of note, dingos, like domestic dogs, are not a single colour [https://doi.org/10.1111/jzo.12875]. This existing diversity is consistent with the author's statement of lines 198-199 for the timing of colour variation (depending on accepted dingo age 3,500-8,000 ybp), but lines 233-234 may misrepresent the dingo group as only having the dominant yellow colour.

The text has been corrected as suggested (line 233).

Detailed notes.
Figure 2a. Please add genomic coordinates for the SINE and LINE elements, or if not possible, please indicate their size and orientation. This may make Fig 2a too busy, but this information should be reported in some fashion in the manuscript and then linked back to this figure. This could help the reader understand why HCP3 and 5, with intact exon 2 are loss of function haplotypes. Are the duplicate copies of SINE C2A1_CF full length? This could impact the regulatory potential of the element.
Can the authors place the known SINE element from Dreger et al. 2011 in the context of their figure 2a? This element is genotyped in the paper but its relationship to the newly reported SINEs is not clear.

Figure 2 has been updated to provide genomic context and as the reviewer suggested we have added specific HGVS description of the variants. We emphasize that all our newly reported variation is occurring within 1.5 kb of the transcription start sites. We also added Fig 3 and a description of our genetic evidence that HCP3,4 and 5 are all loss of function.

Figure 2b could be misleading. At first glance it appears that the semi-quantitative expression was measured in the number of individuals (N) under the dog illustrations. This is not the case. Rather these numbers seem to reflect the 352 individuals genotyped from supplementary Table 5. This is important, as there are very few data points to support the transcription analysis (only one data point for VP1 in Extended Data Fig1). The authors report Extended Data Fig1 in their figure description, but difference between RNA seq results and genotyping could be clearer.

We agree, and have removed the number of genotyped animals from Figure 2 so as to avoid confusion. The genotype-phenotype association is now separately given in Table 1 in the main text in addition to the more detailed data in the Supplementary tables. We also have been more explicit regarding the numbers of dogs and their phenotypes from which RNA-seq data was available (legend to Supplementary Fig. 1, Supplementary Table 8, line 66 and in the methods).

The authors state that HCP3-5 are loss of function haplotypes, as evidenced by the lack of transcripts identified from the RNA-seq. Was it possible to extract DNA from the tissues samples at the same time as RNA to confirm no other LOF variants in the transcripts, and so assure the reader that the LOF is from the regulatory module?

Our WGS analysis shows conclusively that there are no coding sequence changes associated with HCP3, 4, or 5. We have clarified the reasoning behind our conclusion that loss-of-function in HCP3, 4, and 5 is due to structural variation close to the promoter (lines 100-109).

Why did the authors discount the third ASIP transcription start site in their analysis? It is illustrated in

Extended Data Figure 2, but is not shown in Figs 2 or 3 in the main text. Do the authors not believe it is a true, or translated transcript? Could it have had the potential to influence the colours described here?

As noted above, transcripts from the third promoter, which lies ~ 16 kb upstream from the VP, did not vary in abundance and/or structure in our dataset, and therefore we do not know the function of the third promoter, if any. This point is stated explicitly in the revised manuscript (line 76).

Figure 3. Is the Tibetan wolf (Supplementary Table 10) part of fig 3b? Extended data figures 4 and 5 show it as being part of this analysis. If that is so, is this analysis based solely on the individuals in Supplementary Table 9 (line 123), or are individuals in Supplementary Table 10 also included?

The phylogenetic analysis (Fig. 4b, Supplementary fig. 3 and 4, Supplementary Table 9) includes one (SAMN03653004) of the eight Tibetan wolves in Supplementary Table 11.

Line 126. Do you mean Fig 3b and 3c here?

The reference on line 126 has been modified to Fig. 4a-c. Fig. 4a-b show haplotype similarity, and Fig. 4c shows the coat color of arctic wolves from Ellesmere Island and Greenland.

Line 128. Which polymorphic site is variable between grey wolves? Extended Data Table 2 does to report that information. In my copy of Fig2 some genotypes appear to be missing (white), but I assume the variant being referred to is closer to the 23.33 Mb end.

The polymorphic site in arctic wolves is upstream of the VP. Sequence coordinates and a reference to Fig. 4a have been added to the main text.

Extended Data Table 2. What is a Yana wolf?

The Yana wolf is an ancient DNA sample discussed subsequently in the main text and included in Fig. 5a and Supplementary Table 11. A footnote was added to Supplementary Table 10 for clarification.

Supplementary Table 1. Shaded yellow and dominant yellow have the same accession number but represent different haplotypes. Is one of these incorrect?

We revised Supplementary Table 1 and give now HGVS variant designations. In this table, we separated the ventral promoter from the hair cycle promoter modules. The accession numbers only refer to the hair cycle promoter module. We revised the header of the column to make this clearer.

Lines 76-77, 337-339 and Supplementary Table 2. 77 samples are described in the text and reported in the table, but the designations do not match. Please fix. Under “color” in the table, 5 wolves are

described as “wolf”, not a coat colour. These “n.d.” individuals seem to be merged with the agouti individuals in the text description.

We believe that you mean Sup Table 3. We did make the assumption that the wolves were Agouti in coat pattern- this has been changed in the Supplementary Table 3.

Lines 337-339. How many genomic variants were detected from the WGS data of 77 dogs and wolves? How were these dissected for segregation pattern to coat colour? Were only promoter regions considered for colour outcome, or was the 30 kb gene space considered? Were variants phased?

We used homozygotes rather than phasing and only investigated the 2 kb upstream of the transcription start sites. It was apparent that there was variation there that segregated within these samples with color pattern. This variation is both necessary and sufficient to explain the different pattern phenotypes.

Lines 311-328. Skin biopsies and RNA sequencing. Six skin biopsies are described, two per individual. Was each of the libraries barcoded before sequencing? If yes, can the authors report the range of reads per sample, rather than the average?

All skin samples were processed individually and all libraries were barcoded individually. This has been added to the methods section. We also added the number of sequence reads for each library to Supplementary Table 8.

Lines 330-335. Transcript coordinates. The published sample, SRX1884098, is mentioned as being retrieved during the whole transcript sequencing phase. What were the transcript coordinates for this sample? If it was not used during the alignment phase, what was its purpose?

We did not use this sample for the analysis of ventral specific expression since we did not know where on the dog the sample was acquired. It was used to define transcript coordinates including transcription start sites and for the analysis of hair-cycle specific expression.

In Supplementary Table 2, it is not noted which gene models derived from which samples. If this is known, can the authors please report the findings? How do the three new transcripts relate to the provisional RefSeq transcript, NM_001007263.1?

We have made a change in the methods (line 389) and in Figure 2 indicating the overlap between the transcripts that we identified and NCBI annotated transcripts. For all transcripts, our first exons started a few nucleotides up- or downstream of the NCBI annotations, but were otherwise identical. The RefSeq transcript NM_001007263.1 corresponds to the transcript expressed from the ventral promoter.

Lines 341-342. “…used for visual inspection of the promoter regions based on the transcripts identified

in the RNA sequencing data." Does this include the three ASIP transcripts in Supplementary Table 2, or only the two that are described in more detail throughout the manuscript?

Visual inspection was performed for all three promoter regions. Genotypes at variants near the most proximal (5'-) promoter did not associate with the coat patterns as defined in Fig 1. This has been clarified in the methods section (line299) and main text (Line 76).

Why is Supplementary Table 5 (line 346) mentioned before Supplementary Table 4 (line 353) in the methods? Can these numbers be swapped?

These have been changed as suggested.

Lines 365-374 Genotyping. Variable markers from three publications are mentioned in the genotyping section, but their relationship to the newly discovered elements is not clear. Please update Supplementary Table 4 to include the HGVS location of all new targets and perhaps present these in physical location order.

We changed the order of presentation as suggested (this is now Suppl. Table 5). We now consistently give HGVS designations for all tested markers.

Lines 412. What region was considered in the haplotype reconstruction? PRJEB32865 is not publicly available. Is this being updated? From SRA, "No public data is linked to this project." PRJNA448733 is "… 722 genomes sequenced via WGS containing various wild canids, dingo, and domesticated dogs." Are 722 considered at this stage?

PRJEB32865 is available in ENA (European Nucleotide Archive). In Supplemental Table 3 there is a column I which indicates which samples were used for the haplotype construction.

Supplementary Table 3. How was the colour of each individual assigned? Was this in the same fashion as for Supplementary Table 5 (see text lines 305-308)?

The methods (lines 411) now clarifies how the pattern type of all individuals was determined.

Supplementary Tables 4/5/7. From the title description, "…Genotypes at previously used diagnostic markers (5,6,7) are also given." Does 5,6,7 indicate references? From which reference section, there are three to chose from. Please link the references to each table, with PMIS, doi or extended identifier.

These references are now specified with digital object identifiers (doi).

Supplementary Table 5 notes the genotyping of "Black and tan insertion: NC_006606.3:g.23365284_23365285insHQ910237; N=no insertion, I=insertion" and gives the result over two boxes (assume one for each chromosome). How is the reader to interpret the variant

nomenclature? Is the full 1113bp of HQ910237 genotyped, or just the sine element? Supplementary Table 4 seems to indicate only 160bp of a SINE element. Suggest the authors reformat their description to reflect HGVS (e.g. NC_006606.3:g.23365284_23365285insHQ910237:X_Y, where X and Y are the relevant bases of HQ910237).

Thank you for spotting this error. We have revised the variant designations in Supplementary tables 4 and 5 accordingly, and note that the SINE insertion is ~240 bp in size with some variability depending on the length of the SINE-associated polyA-tract.

Supplementary Table 8 notes "ASIP mean coverage depth", but not every sample has a value. Could the missing samples not be aligned? Seems unlikely as some missing samples were retrieved as aligned reads.

The mean coverage depth values in Supplementary Table 9 are now complete.

Supplementary Table 10. What is the difference between no entry in the table and n.d.? What does n.d. mean? What is "Collection site coordinates"? What is "?" in "HCP Repeat Elements". Does SAMN14210384 have true deletions or are is this an issue to do with the age of the sample? If true deletion, please state break points.

"n.d." indicates a locus at which a genotype could not be determined from sequence directly. A footnote was added to the table with an explanation. All genotype cells are populated with a genotype or "n.d."

The column header "Collection site coordinates" has been changed to "Collection site GPS coordinates (latitude, longitude)".

The Zhokov dog sample (SAMN14210384) is single-end sequenced. The HCP3/4-specific SINE-C1A_cf (Fig. 4D) cannot be detected from single-end sequence and is inferred from an identical haplotype observed in modern Black Back dogs. The "?" has been replaced with a footnoted explanation in Table S10. The deletion is inferred from the absence of reads spanning the deletion interval and from split reads at the breakpoint junctions. The deletion breakpoints are identical to those observed in modern Black Back dogs (Table S5) and coordinates are now provided as a footnote in Table S10.

Extended Data Fig 2. The figure would benefit from the annotation of genomic positions, For example, 70kb to RALY Dup. 70kb from where? What is the size of the blocks considered in the haplotype figure? How many individuals are considered in each colour class? I estimate 7 in AG, and this matches Sup Table 3, but it would help to label the figure as DY is 8, BS is 5 etc, but the haplotype blocks are very similar in size. The colour "wolf" is indicated to be used in this section from Sup Table 3, but I do not see it in the figure. What is a "more primitive breed" as described by the authors in this figure legend? Were they genotyped in this paper, or were they used in the publications referred to in this work?

These concerns have been addressed by revisions to Fig. 2a and Supplementary Fig. 2.

The color coding scheme is: yellow homozygous reference, blue homozygous alternate, and grey heterozygote (the legend has been corrected). All the SNVs are shown. The number of dogs by color is 8 AG, 7 BB, 5 BS, 8 DY and 6 SY as shown in Supplementary Table 3. The reference to "wolf color" was an error (thank you for pointing that out) and has been removed. The primitive breeds include Alaskan Malamutes, Sloughi, Jamthund, Pekingese, Chow, Siberian husky and Basenji.

In the supplementary tables but not the text, the authors discuss the incorrect segregation of commercial markers to phenotype. Seems strange to use the terms "commercial" here, but refer to the publications that identified them in the rest of the text.

We changed this and removed the "commercial" terminology throughout.

Be consistent with naming. For example, Fig. 3B or Fig. 3b in the descriptions of Extended Figures 4 and 5. Fig. 2B or Fig. 2b? Both are used in the text. Is the reference canFam3.1 or CanFam3.1? Both are used in the text.

CanFam3.1 and we have corrected it.

Check that correct image table is being cited. For example, Supplementary Table 2 refers to FigS2, but there is no FigS2 in the manuscript. Suspect this is for Extended Figure 2.

This has been corrected.

Decision Letter, first revision:

12th May 2021

Dear Danika,

Thank you for submitting your revised manuscript "Dog color patterns explained by modular promoters of ancient canid origin" (NATECOLEVOL-210212841A). It has now been seen again by the original reviewers and their comments are below. The reviewers find that the paper has improved in revision, and therefore we will be happy in principle to publish it in Nature Ecology & Evolution, pending minor revisions to satisfy the reviewers' final requests and to comply with our editorial and formatting guidelines.

If the current version of your manuscript is in a PDF format, please email us a copy of the file in an

editable format (Microsoft Word or LaTex)-- we can not proceed with PDFs at this stage.

We are now performing detailed checks on your paper and will send you a checklist detailing our editorial and formatting requirements in about a week. Please do not upload the final materials and make any revisions until you receive this additional information from us.

Thank you again for your interest in Nature Ecology & Evolution. Please do not hesitate to contact me if you have any questions.

[REDACTED]

Reviewer #1 (Remarks to the Author):

The authors have dealt with my comments appropriately. I have no further comments. It's a very nice piece of work.

Reviewer #2 (Remarks to the Author):

The authors have addressed all my concerns. I don't have any additional comments.

Very nice manuscript!

Reviewer #3 (Remarks to the Author):

My concerns from review one have been addressed in full.
I have only minor formatting comments listed below. These do not detract from the suitability of this work for publication.

Minor
Supplementary Table 1. Check nomenclature for chromosome 24 of CanFam3.1. Should be NC_006606.3 not NC_00606.3

Supplementary Table 3. Includes wolves, may change description to “samples” not “dogs”.

Supplementary Table 10.Yanna wolf in fig 4d, not 4a.

Line 76-79. If WGS from 77 samples was used to determine the 2 VP and 5 HCP haplotypes (See also Supplementary Table 3), why are only a subset of these (n=33) used to plot Supplementary Fig 2? It could be appropriate to refer to this figure in this paragraph (lines 75-84) as well as the next, where genotyping is discussed.

Supplementary Table 9. Should the scientific name for the New Guinea Singing Dog be Canis dingo hallstromi, or Canis hallstromi. I’m not familiar with the literature that classes them in the same block as the Australian dingo, Canis lupus dingo

Line 77. What is the definition of ASIP locus? Is this the coding fraction of the gene? Which element is homozygous, that then allows for the dissection of VP and HCP haplotypes? Note, in Supplementary Fig 5, the ASIP locus spans the 48 kb VP and the 16kn HCP and coding exons.

Lines 112-113. Similar to issue with line 77. The authors say that they compare 18 homozygous dogs to 10 wolves. The figure accompanying the text (Fig 4a) shows variation across this region in the dogs. Could the authors add a bit more definition to this sentence to indicate which element was homozygous?

Line 334-335. As per line 77. What is the genomic span of the ASIP haplotype used to visualise haplotypes?

Our ref: NATECOLEVOL-210212841A

14th May 2021

Dear Dr. Bannasch,

Thank you for your patience as we've prepared the guidelines for final submission of your Nature Ecology & Evolution manuscript, "Dog color patterns explained by modular promoters of ancient canid origin" (NATECOLEVOL-210212841A). Please carefully follow the step-by-step instructions provided in the attached file, and add a response in each row of the table to indicate the changes that you have made. Please also check and comment on any additional marked-up edits we have proposed within the text. Ensuring that each point is addressed will help to ensure that your revised manuscript can be swiftly handed over to our production team.

We would like to start working on your revised paper, with all of the requested files and forms, as soon as possible (preferably within two weeks). Please get in contact with us immediately if you anticipate it taking more than two weeks to submit these revised files.

When you upload your final materials, please include a point-by-point response to any remaining reviewer comments.

If you have not done so already, please alert us to any related manuscripts from your group that are under consideration or in press at other journals, or are being written up for submission to other journals (see: https://www.nature.com/nature-research/editorial-policies/plagiarism#policy-on-duplicate-publication for details).

In recognition of the time and expertise our reviewers provide to Nature Ecology & Evolution's editorial process, we would like to formally acknowledge their contribution to the external peer review of your

manuscript entitled "Dog color patterns explained by modular promoters of ancient canid origin". For those reviewers who give their assent, we will be publishing their names alongside the published article.

Nature Ecology & Evolution offers a Transparent Peer Review option for new original research manuscripts submitted after December 1st, 2019. As part of this initiative, we encourage our authors to support increased transparency into the peer review process by agreeing to have the reviewer comments, author rebuttal letters, and editorial decision letters published as a Supplementary item. When you submit your final files please clearly state in your cover letter whether or not you would like to participate in this initiative. Please note that failure to state your preference will result in delays in accepting your manuscript for publication.

Cover suggestions

As you prepare your final files we encourage you to consider whether you have any images or illustrations that may be appropriate for use on the cover of Nature Ecology & Evolution.

Covers should be both aesthetically appealing and scientifically relevant, and should be supplied at the best quality available. Due to the prominence of these images, we do not generally select images featuring faces, children, text, graphs, schematic drawings, or collages on our covers.

We accept TIFF, JPEG, PNG or PSD file formats (a layered PSD file would be ideal), and the image should be at least 300ppi resolution (preferably 600-1200 ppi), in CMYK colour mode.

If your image is selected, we may also use it on the journal website as a banner image, and may need to make artistic alterations to fit our journal style.

Please submit your suggestions, clearly labeled, along with your final files. We'll be in touch if more information is needed.

Nature Ecology & Evolution has now transitioned to a unified Rights Collection system which will allow our Author Services team to quickly and easily collect the rights and permissions required to publish your work. Approximately 10 days after your paper is formally accepted, you will receive an email in providing you with a link to complete the grant of rights. If your paper is eligible for Open Access, our Author Services team will also be in touch regarding any additional information that may be required to arrange payment for your article.

Please note that <i>Nature Ecology & Evolution</i> is a Transformative Journal (TJ). Authors may publish their research with us through the traditional subscription access route or make their paper immediately open access through payment of an article-processing charge (APC). Authors will not be required to make a final decision about access to their article until it has been accepted. Find out more about Transformative Journals

<B>Authors may need to take specific actions to achieve compliance with funder and institutional open access mandates. For submissions from

January 2021, if your research is supported by a funder that requires immediate open access (e.g. according to Plan S principles) then you should select the gold OA route, and we will direct you to the compliant route where possible. For authors selecting the subscription publication route our standard licensing terms will need to be accepted, including our self-archiving policies. Those standard licensing terms will supersede any other terms that the author or any third party may assert apply to any version of the manuscript.

Please note that you will not receive your proofs until the publishing agreement has been received through our system.

For information regarding our different publishing models please see our Transformative Journals page. If you have any questions about costs, Open Access requirements, or our legal forms, please contact ASJournals@springernature.com.

Please use the following link for uploading these materials:
[REDACTED]

If you have any further questions, please feel free to contact me.

[REDACTED]

Reviewer #1:
Remarks to the Author:
The authors have dealt with my comments appropriately. I have no further comments. It's a very nice piece of work.

Reviewer #2:
Remarks to the Author:
The authors have addressed all my concerns. I don't have any additional comments.

Very nice manuscript!

Reviewer #3:
Remarks to the Author:
My concerns from review one have been addressed in full.
I have only minor formatting comments listed below. These do not detract from the suitability of this work for publication.

Minor
Supplementary Table 1. Check nomenclature for chromosome 24 of CanFam3.1. Should be NC_006606.3 not NC_00606.3

Supplementary Table 3. Includes wolves, may change description to “samples” not “dogs”.

Supplementary Table 10.Yanna wolf in fig 4d, not 4a.

Line 76-79. If WGS from 77 samples was used to determine the 2 VP and 5 HCP haplotypes (See also Supplementary Table 3), why are only a subset of these (n=33) used to plot Supplementary Fig 2? It could be appropriate to refer to this figure in this paragraph (lines 75-84) as well as the next, where genotyping is discussed.

Supplementary Table 9. Should the scientific name for the New Guinea Singing Dog be Canis dingo hallstromi, or Canis hallstromi. I’m not familiar with the literature that classes them in the same block as the Australian dingo, Canis lupus dingo

Line 77. What is the definition of ASIP locus? Is this the coding fraction of the gene? Which element is homozygous, that then allows for the dissection of VP and HCP haplotypes? Note, in Supplementary Fig 5, the ASIP locus spans the 48 kb VP and the 16kn HCP and coding exons.

Lines 112-113. Similar to issue with line 77. The authors say that they compare 18 homozygous dogs to 10 wolves. The figure accompanying the text (Fig 4a) shows variation across this region in the dogs. Could the authors add a bit more definition to this sentence to indicate which element was homozygous?

Line 334-335. As per line 77. What is the genomic span of the ASIP haplotype used to visualise haplotypes?

Author Rebuttal, first revision:

Reviewer #1:
Remarks to the Author:
The authors have dealt with my comments appropriately. I have no further comments. It's a very nice piece of work.

Reviewer #2:
Remarks to the Author:
The authors have addressed all my concerns. I don't have any additional comments.

Very nice manuscript!

Reviewer #3:
Remarks to the Author:
My concerns from review one have been addressed in full.
I have only minor formatting comments listed below. These do not detract from the suitability of this work for publication.

Minor

All minor errors have been changed as suggested. Explanations when needed are included in italics below each query.

Supplementary Table 1. Check nomenclature for chromosome 24 of CanFam3.1. Should be NC_006606.3 not NC_00606.3

Supplementary Table 3. Includes wolves, may change description to "samples" not "dogs".

Supplementary Table 10.Yanna wolf in fig 4d, not 4a.

This is now Fig 5a

Line 76-79. If WGS from 77 samples was used to determine the 2 VP and 5 HCP haplotypes (See also Supplementary Table 3), why are only a subset of these (n=33) used to plot Supplementary Fig 2? It could be appropriate to refer to this figure in this paragraph (lines 75-84) as well as the next, where genotyping is discussed.

Specific individual animals that were homozygous were chosen for Supplementary Figure 2 as indicated in the methods section under the heading ***Haplotype Construction****. This data is introduced later after the genotyping since this is the order that we performed the analysis.*

Supplementary Table 9. Should the scientific name for the New Guinea Singing Dog be Canis dingo hallstromi, or Canis hallstromi. I'm not familiar with the literature that classes them in the same block as the Australian dingo, Canis lupus dingo

Line 77. What is the definition of ASIP locus? Is this the coding fraction of the gene? Which element is homozygous, that then allows for the dissection of VP and HCP haplotypes? Note, in Supplementary Fig 5, the ASIP locus spans the 48 kb VP and the 16kn HCP and coding exons.

The definition of the locus includes the first two non-coding exons that we are studying (VP and HCP). We utilized dogs likely to be homozygous based on the breed and phenotypes. The homozygous elements included VP, HVP and coding exons based on visual inspection. This is consistent with Sup. Figure 5.

Lines 112-113. Similar to issue with line 77. The authors say that they compare 18 homozygous dogs to 10 wolves. The figure accompanying the text (Fig 4a) shows variation across this region in the dogs. Could the authors add a bit more definition to this sentence to indicate which element was homozygous?

Added that these animals were homozygous at the structural variants at VP and HCP as well as the coding exons.

Line 334-335. As per line 77. What is the genomic span of the ASIP haplotype used to visualise haplotypes?

The homozygous elements included VP, HVP and coding exons based on visual inspection. This has been added to the text.

Final Decision Letter:

1st July 2021

Dear Danika,

We are pleased to inform you that your Article entitled "Dog color patterns explained by modular promoters of ancient canid origin", has now been accepted for publication in Nature Ecology & Evolution.

Before your manuscript is typeset, we will edit the text to ensure it is intelligible to our wide readership and conforms to house style. We look particularly carefully at the titles of all papers to ensure that they are relatively brief and understandable.

The subeditor may send you the edited text for your approval. Once your manuscript is typeset you will receive a link to your electronic proof via email, with a request to make any corrections within 48 hours. If you have queries at any point during the production process then please contact the production team at rjsproduction@springernature.com. Once your paper has been scheduled for online publication, the Nature press office will be in touch to confirm the details.

Acceptance of your manuscript is conditional on all authors' agreement with our publication policies (see www.nature.com/authors/policies/index.html). In particular your manuscript must not be published elsewhere and there must be no announcement of the work to any media outlet until the publication date (the day on which it is uploaded onto our web site).

Please note that <i>Nature Ecology & Evolution</i> is a Transformative Journal (TJ). Authors may publish their research with us through the traditional subscription access route or make their paper immediately open access through payment of an article-processing charge (APC). Authors will not be required to make a final decision about access to their article until it has been accepted. Find out more about Transformative Journals

<B>Authors may need to take specific actions to achieve compliance with funder and institutional open access mandates. For submissions from January 2021, if your research is supported by a funder that requires immediate open access (e.g. according to Plan S principles) then you should select the gold OA route, and we will direct you to the compliant route where possible. For authors selecting the subscription publication route our standard licensing terms will need to be accepted, including our self-archiving policies. Those standard licensing terms will supersede any other terms that the author or any third party may assert apply to any version of the manuscript.

In approximately 10 business days you will receive an email with a link to choose the appropriate publishing options for your paper and our Author Services team will be in touch regarding any additional information that may be required.

You will not receive your proofs until the publishing agreement has been received through our system.

If you have any questions about our publishing options, costs, Open Access requirements, or our legal forms, please contact ASJournals@springernature.com

An online order form for reprints of your paper is available at https://www.nature.com/reprints/author-reprints.html. All co-authors, authors' institutions and authors' funding agencies can order reprints using the form appropriate to their geographical region.

We welcome the submission of potential cover material (including a short caption of around 40 words) related to your manuscript; suggestions should be sent to Nature Ecology & Evolution as electronic files (the image should be 300 dpi at 210 x 297 mm in either TIFF or JPEG format). Please note that such pictures should be selected more for their aesthetic appeal than for their scientific content, and that colour images work better than black and white or grayscale images. Please do not try to design a cover with the Nature Ecology & Evolution logo etc., and please do not submit composites of images related to your work. I am sure you will understand that we cannot make any promise as to whether any of your suggestions might be selected for the cover of the journal.

You can now use a single sign-on for all your accounts, view the status of all your manuscript submissions and reviews, access usage statistics for your published articles and download a record of your refereeing activity for the Nature journals.

natureresearch

To assist our authors in disseminating their research to the broader community, our SharedIt initiative provides you with a unique shareable link that will allow anyone (with or without a subscription) to read the published article. Recipients of the link with a subscription will also be able to download and print the PDF.

You can generate the link yourself when you receive your article DOI by entering it here: <a href="http://authors.springernature.com/share">http://authors.springernature.com/share<a>.

[REDACTED]

P.S. Click on the following link if you would like to recommend Nature Ecology & Evolution to your librarian http://www.nature.com/subscriptions/recommend.html#forms

** Visit the Springer Nature Editorial and Publishing website at www.springernature.com/editorial-and-publishing-jobs for more information about our career opportunities. If you have any questions please click here.**
